# Early-life exposure to the Great Chinese Famine and gut microbiome disruption across adulthood for type 2 diabetes: three population-based cohort studies

**DOI:** 10.1186/s12916-023-03123-y

**Published:** 2023-11-01

**Authors:** Wanglong Gou, Huijun Wang, Xin-yi Tang, Yan He, Chang Su, Jiguo Zhang, Ting-yu Sun, Zengliang Jiang, Zelei Miao, Yuanqing Fu, Hui Zhao, Yu-ming Chen, Bing Zhang, Hongwei Zhou, Ju-Sheng Zheng

**Affiliations:** 1 Center for Intelligent Proteomics, Westlake Laboratory of Life Sciences and Biomedicine, Hangzhou, China; 2 School of Life Sciences, Westlake University, Hangzhou, China; 3 National Institute for Nutrition and Health, Chinese Center for Disease Control and Prevention, Beijing, China; 4 Key Laboratory of Trace Element Nutrition, National Health Commission, Beijing, China; 5 The Third Affiliated Hospital of Sun Yat-Sen University, Guangzhou, China; 6 Microbiome Medicine Center, Division of Laboratory Medicine, Zhujiang Hospital, Southern Medical University, Guangzhou, China; 7 Guangdong Provincial Key Laboratory of Food, Nutrition and Health, Department of Epidemiology, School of Public Health, Sun Yat-Sen University, Guangzhou, China; 8 Institute of Basic Medical Sciences, Westlake Institute for Advanced Study, Hangzhou, China

**Keywords:** Famine, Gut microbiome, Type 2 diabetes, DOHaD

## Abstract

**Background:**

The early life stage is critical for the gut microbiota establishment and development. We aimed to investigate the lifelong impact of famine exposure during early life on the adult gut microbial ecosystem and examine the association of famine-induced disturbance in gut microbiota with type 2 diabetes.

**Methods:**

We profiled the gut microbial composition among 11,513 adults (18–97 years) from three independent cohorts and examined the association of famine exposure during early life with alterations of adult gut microbial diversity and composition. We performed co-abundance network analyses to identify keystone taxa in the three cohorts and constructed an index with the shared keystone taxa across the three cohorts. Among each cohort, we used linear regression to examine the association of famine exposure during early life with the keystone taxa index and assessed the correlation between the keystone taxa index and type 2 diabetes using logistic regression adjusted for potential confounders. We combined the effect estimates from the three cohorts using random-effects meta-analysis.

**Results:**

Compared with the no-exposed control group (born during 1962–1964), participants who were exposed to the famine during the first 1000 days of life (born in 1959) had consistently lower gut microbial alpha diversity and alterations in the gut microbial community during adulthood across the three cohorts. Compared with the no-exposed control group, participants who were exposed to famine during the first 1000 days of life were associated with consistently lower levels of keystone taxa index in the three cohorts (pooled beta − 0.29, 95% CI − 0.43, − 0.15). Per 1-standard deviation increment in the keystone taxa index was associated with a 13% lower risk of type 2 diabetes (pooled odds ratio 0.87, 95% CI 0.80, 0.93), with consistent results across three individual cohorts.

**Conclusions:**

These findings reveal a potential role of the gut microbiota in the developmental origins of health and disease (DOHaD) hypothesis, deepening our understanding about the etiology of type 2 diabetes.

**Supplementary Information:**

The online version contains supplementary material available at 10.1186/s12916-023-03123-y.

## Background

The developmental origins of health and disease (DOHaD) hypothesis suggests that adverse exposures during early life, particularly in utero, may substantially influence the later-life health and disease status [1]. Indirect support for this hypothesis comes from studies showing consistent associations of early-life famine exposure with increased risk of type 2 diabetes [2–6].

There have been several proposed theories to support the DOHaD concept and indicate the potential mechanism, including the thrifty gene [7], bet-hedging, fetal predictive adaptive response [8], and drifty phenotype hypotheses [9]. The gut microbiota is considered as a fundamental part of human physiology, contributing to the regulation of host health [10]. Gut microbiota aberrations have been associated with multiple metabolic disorders, such as type 2 diabetes [11–13]. Analogous to DOHaD, the early life, especially the first 1000 days (from conception to 2 years of age) of life, is critical for gut microbiota establishment and development [14]. Environmental insults during the period can disrupt the optimal succession of the gut microbiota [15]. The above evidence leads us to propose the hypothesis that the gut microbiome may be a key component contributing to the DOHaD concept.

China experienced the Great Famine during 1959–1961. Famine, as a natural experiment, provides a unique opportunity to examine the impact of early-life adverse exposure on the adult and later-life gut microbiota and the role of the gut microbiota in the DOHaD. Therefore, using three independent cohorts involving 11,513 participants covering 16 major provinces/megacities across China, we aimed to investigate the longitudinal associations of famine exposure during early life with the adult gut microbial diversity, community structure, and keystone taxa. As a secondary objective, we aimed to examine the association of famine-induced disturbance in the gut microbial ecosystem with type 2 diabetes.

## Methods

### Study cohorts

The present study was based on the gut microbiome cohort consortium: the Westlake Gut Project [16], including three independent Chinese cohorts: Guangzhou Nutrition and Health Study (GNHS) [17], Guangdong Gut Microbiome Project (GGMP) [18], and China Health and Nutrition Survey (CHNS) [19]. The GNHS is a prospective cohort study conducted in the Guangdong province. It employed a non-probability sampling method to recruit participants who had resided in Guangzhou city for at least 5 years. Ultimately, between the years 2008 and 2013, GNHS recruited 4048 participants [17]. During subsequent follow-up visits from 2014 to 2018, a subset of 1935 participants provided stool samples for the measurement of 16S rRNA. After excluding the participants with antibiotics used within the month preceding stool collection or without information about the date of birth or with low depth of sequencing reads (< 5000), 1920 participants (age 64.9 ± 5.9 years, mean ± SD) from the GNHS were included in the present study (Additional file 1: Fig. S1).

The GGMP was a cross-sectional cohort study conducted during 2015 and 2016. The study participants were selected based on a stratified random sampling method. Ultimately, the study included a total of 7009 participants from 14 districts within Guangdong province [18]. All the participants within the GGMP had their stool samples collected for the measurement of 16S rRNA. After adopting the same inclusion and exclusion criteria as used in GNHS, 6560 GGMP participants were included in the present study (age 52.7 ± 14.8 years, mean ± SD).

The CHNS is a national longitudinal cohort study in China. Participants in CHNS were selected through a random process from 15 provinces across the country. It is important to mention that Guangdong province was not included in the CHNS study. Within each of these provinces, a multistage, random cluster process was used to draw participants [19]. CHNS rounds were completed in 1989, 1991, 1993, 1997, 2000, 2004, 2006, 2009, 2011, 2015, and 2018. During the 2015 round, stool samples from a total of 3248 participants were collected with subsequent measurements of 16S rRNA [19]. After adopting the same inclusion and exclusion criteria as used in the GNHS, in the present study, we included 3033 CHNS participants from 15 provinces or megacities (age 51.6 ± 12.7 years, mean ± SD).

### Measurement of metadata

Demographic and medication data were collected by questionnaires during the face-to-face questionnaire interviews. The demographic data of this study included age, sex, date of birth, and the average income of all household members. Medication data included the use of antibiotics, hypoglycemic drugs, and hypolipidemic drugs. Anthropometric factors, including height and weight, were measured on-site by the trained staff. Blood samples were collected by registered nurses following an overnight fast.

In the GNHS cohort, high-performance liquid chromatography was used to measure glycated hemoglobin (HbA1c) using the Bole D-10 Hemoglobin A1c Program on a Bole D-10 Hemoglobin Testing System; fasting glucose was determined enzymatically on a Hitachi 7600–010 automated analyzer (Hitachi, Tokyo, Japan). In the GGMP cohort, fasting glucose and HbA1c were measured on a Hitachi 7600 automatic biochemical analyzer using reagents obtained from Wako Pure Chemical Industries Ltd. at the National CDC of China. In the CHNS cohort, all samples were analyzed in a national central lab in Beijing (medical laboratory accreditation certificate ISO 15189:2007) with strict quality control. Blood glucose levels were measured using a glucose oxidase phenol 4-aminoantipyrine peroxidase kit (Randox, Crumlin, UK) and a Hitachi 7600 Analyzer (Hitachi, Tokyo, Japan); HbA1c was measured via high-performance liquid chromatography system (model HLC-723 G7; Tosoh Corporation, Tokyo, Japan).

### Gut microbiome analyses

Stool samples from each cohort were sequenced in a single batch. Detailed methods for the gut microbiome analyses are provided in Additional file 1: Supplementary methods.

### Assessment of famine exposure

China experienced the Great Famine during 1959–1961. People from all provinces experienced the effects of the famine, resulting in a notable decrease in birth rate and a simultaneous increase in mortality rate across China [20]. Consistent with previous Chinese famine studies [21, 22], we used the birth year of the participants as the basis for the classification of famine exposure. Participants born between 1962 and 1964 were classified as the no-exposed control group, and those born after 1964 were classified as the no-exposed group. Participants born between 1959 and 1961 were classified as the utero exposed group. The utero-exposed group was further divided into three sub-groups to capture their durations of exposure to famine. Specifically, participants who were born in 1959 had been exposed to famine in utero and the first two postnatal years (first 1000 days of life). Participants born in 1960 had been exposed to famine in utero and the first postnatal year. Participants born in 1961 were only exposed to famine in utero. Participants born before 1959 were classified into five exposed groups: infancy and toddler exposed group (born between 1956 and 1958), preschooler exposed group (born between 1953 and 1955), school-aged child exposed group (born between 1947 and 1952), adolescent exposed group (born between 1942 and 1946), and adult exposed group (born before 1942).

### Assessments of type 2 diabetes

In the GNHS and CHNS cohorts, type 2 diabetes cases were ascertained based on fasting blood glucose ≥ 7.0 mmol/L or HbA1c ≥ 47.5 mmol/mol (6.5%) or being currently under medical treatment for type 2 diabetes during the collection of stool samples, according to the American Diabetes Association criteria [23] for the diagnosis of diabetes. In the GGMP study, type 2 diabetes was determined by self-report (confirmed with medical history) or fasting blood glucose ≥ 7.0 mmol/L.

### Statistical methods

#### Comparison of the gut microbial landscape among GNHS, GGMP, and CHNS participants

At the genus level, we used the *vegdist* function in the R package vegan [24] to calculate the gut microbial Bray–Curtis dissimilarity matrix. The pairwise comparisons of the variation in microbial composition between three cohorts (GNHS, GGMP, and CHNS) were determined by PERMANOVA analysis and were visualized using principal coordinate analysis (PCOA). The *P* value was determined by 999 permutations and was further adjusted for multiple testing of pairwise comparison using the Benjamini-Hochberg method (function pairwise Adonis in R package). An adjusted *P* value < 0.05 was considered statistically significant.

#### Famine exposure, gut microbial diversity, and type 2 diabetes

Alpha diversity differences (in SD units) between other groups and no-exposed control group were evaluated by linear regression model, with adjustment for age, sex, BMI, and the use of hypoglycemic and hypolipidemic medications (yes/no for each). The tested alpha diversity indices include observed OTUs, Shannon’s diversity index, Pielou’s evenness, and Faith’s phylogenetic diversity. We pooled the effect estimates from the three cohorts using random-effects meta-analysis. This approach enables a comprehensive analysis that considers the variability among different cohorts.

As sensitivity analyses, firstly, we added a potential technique confounder (sequencing depth), dietary and lifestyle factors, into the covariate list. This adjustment was made to account for their potential impact on the composition of the gut microbiome [25]. Specifically, within the GNHS and CHNS cohorts, the adjusted dietary and lifestyle factors included fruit, vegetable, fish, red meat, dairy, alcohol drinking, and smoking status. In the GGMP cohort, all aforementioned dietary covariates except dairy and fish (not available) were integrated into the model. Secondly, we excluded the participants with type 2 diabetes. Thirdly, we defined participants born after 1978, corresponding to the period after China’s reform and opening-up, as a new reference group, and re-ran the analyses within the GGMP and CHNS cohorts but not GNHS, as there were no participants in the GNHS cohort born after 1978. We also combined the three utero-exposed groups (born in 1959, 1960, and 1961 and with different durations of famine exposure in early life) into one group to assess the association of in utero famine exposure with alpha diversity. As the famine during 1959–1961 affected all provinces of China, it is impossible to find no-exposed controls with a completely matched age structure as the famine births [26]. We therefore combined the participants born in 1962–1964 (no-exposed control group) and 1956–1958 (infancy and toddler exposed group) as a new reference group to balance the age between the no-exposed control group and the utero-exposed groups and re-ran the linear regression analyses.

We examined the cross-sectional association of the aforementioned gut microbial diversity (per SD unit) with type 2 diabetes using logistic regression, with adjustments for age, sex, and BMI. We combined the effect estimates from the three cohorts using random-effects meta-analysis. As a sensitivity analysis, we further added the previously mentioned dietary and lifestyle factors into the covariate list.

#### Famine exposure, gut microbial community structure, and type 2 diabetes

We performed PERMANOVA analysis to evaluate the difference in the overall gut microbial community structure between the no-exposed control group and other groups. The *P* value was determined by 999 permutations. In each of the cohorts, we independently applied principal coordinate analysis to reduce the dimension of the microbial data. The gut microbial genera abundance matrix was represented by the first two principal coordinates of the Bray–Curtis measures. Linear regression was used to evaluate the differences (in SD units) in the two principal coordinates between other groups and the no-exposed control group, with adjustment for the same cofounders as the above alpha diversity analysis.

#### Co-abundance network construction and keystone taxa identification

At the genus level, we filtered the gut microbiota that was detected with < 10% prevalence and used the NetCoMi package [27] to perform microbial co-abundance networks analyses. We used three classical methods (Pearson, SparCC, and SPIEC-EASI) to estimate the microbial correlation matrix and further constructed microbial co-abundance networks. For the Pearson analysis, we used multiplicative imputation [28] to handle zero values and used centered log-ratio (CLR) transformation to move compositional data from the simplex to real space. Correlations with FDR-adjusted *P* values < 0.05 and with a magnitude above 0.3 were selected for further visualization and network analysis. For the SparCC analysis, correlations with FDR-adjusted *P* values < 0.05 and with a magnitude above 0.2 were selected for further visualization and network analysis. SPIEC-EASI was used to infer the conditional dependence between every two microbial taxa. Given that SPIEC-EASI already included node selection strategies, further node filtering was unnecessary. We used a hierarchical agglomeration algorithm [29] to determine clusters of nodes that are highly connected but have a small number of connections to the nodes outside their module.

Here, we used eigenvector centrality to define the keystone in the co-abundance network (node with a centrality value above the empirical 95% quantile in the network). Eigenvector centrality was calculated via eigenvalue decomposition: *Ac* = *λc*, where *λ* denotes the eigenvalues and *c* the eigenvectors of the microbial adjacency matrix A. Eigenvector centrality is then defined as the *i*th entry of the eigenvector belonging to the largest eigenvalue.

Spearman correlation analysis was used to examine the association of keystone taxa and other measured microbial genera with observed OTUs. Correlations with FDR-adjusted *P* values < 0.05 are considered statistically significant.

#### Famine exposure, keystone taxa, and type 2 diabetes

We used a linear regression model to evaluate the difference (in SD unit) in keystone taxa between other groups and the no-exposed control group, with adjustment for age, sex, BMI, and the use of hypoglycemic and hypolipidemic medications (yes/no for each). Here, the gut microbiota data was CLR-transformed. To apply the CLR transformation, zero counts were imputed with a pseudo-count of 1. We constructed a keystone taxa index with the total abundance of the shared keystone taxa that were consistently detected across the three independent cohorts. We also applied the above model to evaluate the association of famine exposure with keystone taxa index. We combined the effect estimates from the three cohorts using random-effects meta-analysis.

Then, we examined the cross-sectional association of CLR-transformed abundance of individual keystone taxa (per SD unit) and keystone taxa index with type 2 diabetes using logistic regression, adjusted for age, sex, and BMI. We combined the effect estimates from the three cohorts using random-effects meta-analysis.

## Results

### Overview of the study participants

**Fig. 1:**
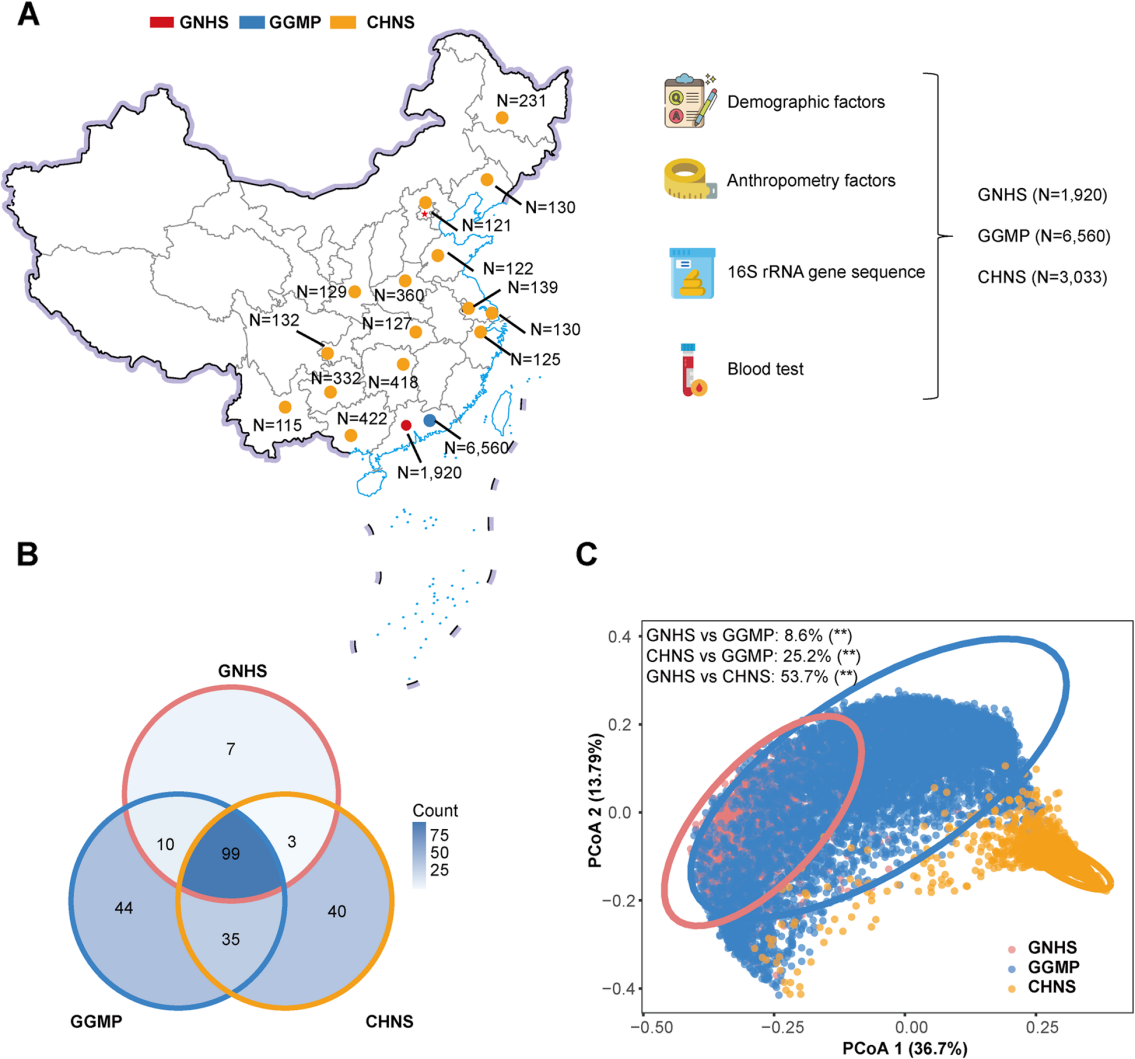
Overview of the study cohorts. **A** Overview of 11,513 participants’ sampling regions. **B** Venn diagram of the number of genera after filtering for the rare genera (< 10% prevalence in the corresponding cohorts) detected in each cohort. **C** Dissimilarities in the gut microbial composition between participants from three cohorts. The pairwise comparisons of the variation in microbial composition among the three cohorts (GNHS, GGMP, and CHNS) were determined by PERMANOVA analyses. The *P* value was determined by 999 permutations and was further adjusted for multiple testing of pairwise comparison using the Benjamini-Hochberg method

A total of 11,513 independent participants (18–97 years) from three Chinese cohorts were included in the present study. Specifically, we included 1920 participants (age 64.9 ± 5.9 years, 67.1% are women) from the GNHS, 6560 (age 52.7 ± 14.8 years, 55.1% are women) from the GGMP, and 3033 (age 51.6 ± 12.7 years, 51.2% are women) from the CHNS. The overall characteristics of the included participants, along with the sample size of each famine exposure group in the three cohorts, were shown in Table 1. The participants included in the present study came from 16 major provinces/megacities in China, and they were either from the same community (GNHS), the same province (GGMP), or from different provinces (CHNS) (Fig. 1A). These cohorts are complementary to each other, given that geographic region is a major confounder in the gut microbiome analysis [18]. A total of 6174 (53.6%) participants have been exposed to the Great Chinese Famine: 595 exposed in utero, 1112 in infancy and toddler, 1227 in preschool, 1832 in school-aged, 778 in adolescence, and 630 in the adult stage. The characteristics of each famine-exposed group and no-exposed group from the three cohorts were shown in Additional file 1: Table S1-S3, respectively.

**Table 1:** Characteristics of the participants included in this study^a^

	GNHS	GGMP	CHNS
Number of participants	1920	6560	3033
Age, years	64.9 (5.9)	52.7 (14.8)	51.6 (12.7)
Women, *n* (%)	1288 (67.1%)	3614 (55.1%)	1554 (51.2%)
BMI, kg/m^2^	23.6 (3.3)	23.4 (3.5)	24.3 (3.9)
Type 2 diabetes case subjects, *n* (%)	268 (14.0%)	553 (8.4%)	368 (12.2%)
Famine exposed groups			
No-exposed control	37 (1.9%)	622 (9.5%)	303 (10.0%)
In utero exposed	54 (2.8%)	366 (5.6%)	175 (5.8%)
Group 1 (born in 1959)	34 (1.8%)	144 (2.2%)	69 (2.3%)
Group 2 (born in 1960)	15 (0.8%)	111 (1.7%)	58 (1.9%)
Group 3 (born in 1961)	5 (0.3%)	111 (1.7%)	48 (1.6%)
Infancy and toddler exposed	341 (17.8%)	512 (7.8%)	259 (8.5%)
Preschooler exposed	425 (22.1%)	502 (7.7%)	300 (9.9%)
School-aged child exposed group	670 (34.9%)	822 (12.5%)	340 (11.2%)
Adolescent exposed group	261 (13.6%)	380 (5.8%)	137 (4.5%)
Adult exposed group	91 (4.7%)	430 (6.6%)	109 (3.6%)
Other no-exposed participants	41 (2.1%)	2926 (44.6%)	1410 (46.5%)

^a^Data are presented as number of participants (%) or mean (SD)

On average, 42,330 ± 12,297 reads in the GNHS, 46,623 ± 20,389 reads in the GGMP and 75,918 ± 11,730 reads in the CHNS were generated. We detected altogether 393 genera in the GNHS cohort, 932 in the GGMP cohort, and 1510 in the CHNS cohort. After filtering for the rare genera (< 10% prevalence in the corresponding cohort), 119, 188, and 177 genera were retained in the three cohorts, respectively. Among the retained genera, most genera detected in the GNHS cohort were also observed in the GGMP (109, 91.6%) and CHNS (102, 85.7%) cohorts (Fig. 1B). We observed relatively small variation between GNHS and GGMP cohorts, but large variation between GNHS and CHNS (Fig. 1C). Furthermore, there is a more pronounced variation between the GGMP and CHNS cohorts (25.2%) compared to the variance between GNHS and GGMP cohorts (8.6%), despite the former employing the same sequencing region. Therefore, these inter-cohort differences were mainly influenced by geographic factors, given that geography has been recognized as a primary determinant of microbial composition [18].

### Exposure to the Great Chinese Famine during the first 1000 days of life is associated with the alterations of gut microbial alpha diversity

**Fig. 2:**
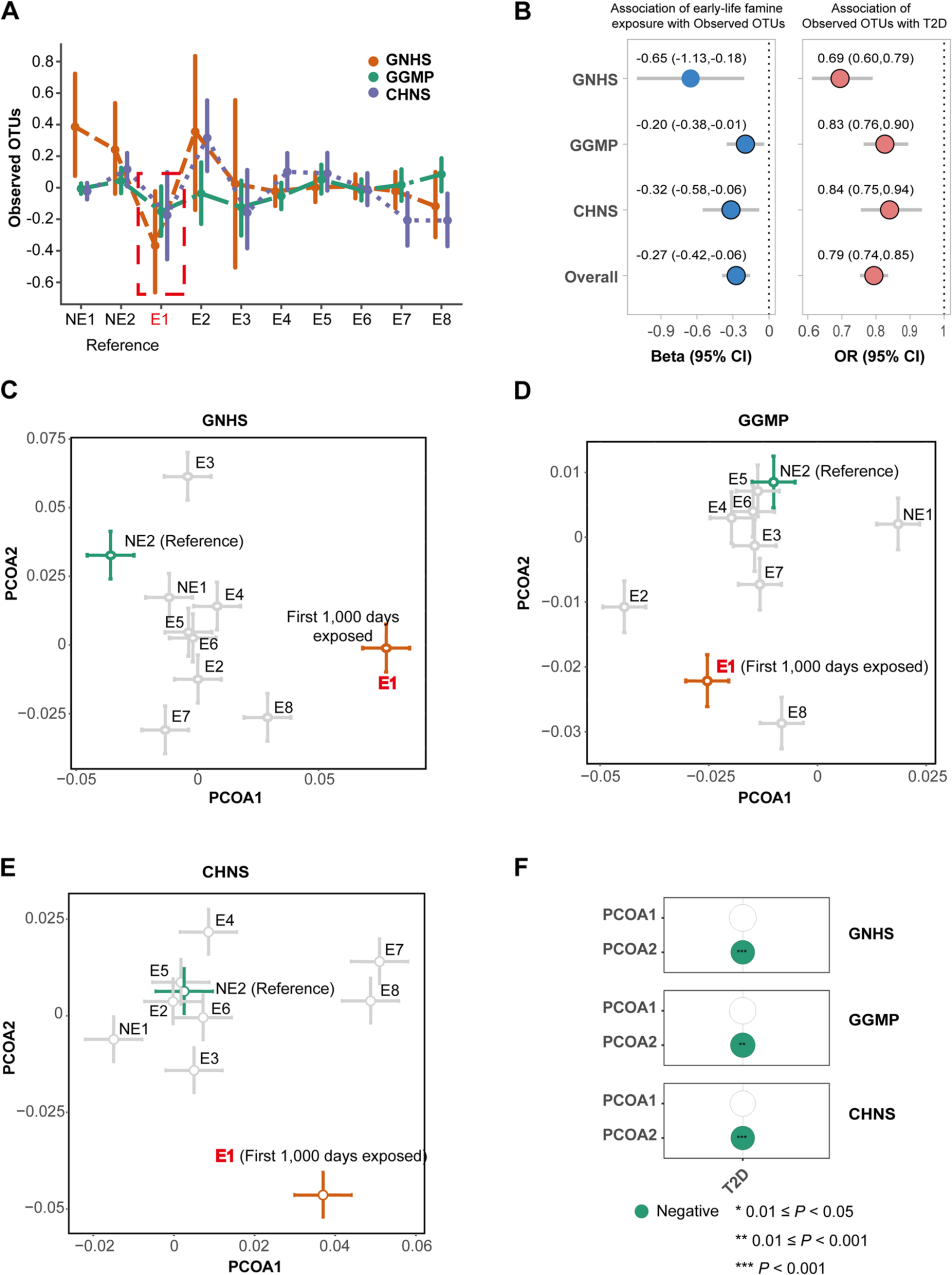
Famine exposure during the first 1000 days of life, alterations of gut microbial diversity, and type 2 diabetes. **A** Distribution of observed OTUs across different groups. Observed OTUs within each cohort were independently *z*-scored. Participants were classified into different groups: the no-exposed group (NE1, born after 1964), no-exposed control group (NE2, born between 1962 and 1964), three in utero exposed groups (E1–E3, born in 1959, 1960, and 1961, respectively), infancy and toddler exposed group (E4, born between 1956 and 1958), preschooler exposed group (E5, born between 1953 and 1955), school-aged child exposed group (E6, born between 1947 and 1952), adolescent exposed group (E7, born between 1942 and 1946), and adult exposed group (E8, born before 1942). Here, participants in the no-exposed control group were used as the reference group. Participants who were exposed to the famine during the first 1000 days of life (E1, born in 1959) were highlighted in red. **B** Left: association of early-life famine exposure with observed OTUs. Linear regression was used to estimate the difference in observed OTUs between other groups and reference group, with adjustment of age, sex, BMI, and the use of hypoglycemic and hypolipidemic medications (yes/no for each). Here, we highlighted the comparation between the E1 and control groups in the three cohorts. Other results were present in the supplemental materials. Right: association of observed OTUs with type 2 diabetes. Logistic regression was used to examine the association of observed OTUs (per SD unit) with type 2 diabetes, adjusted for age, sex, and BMI. We combined the effect estimates from the three cohorts using random-effects meta-analysis. **C** Bray–Curtis-based principal coordinate analysis for the genus-level profiles in the GNHS cohort. The circles and error bars indicate the mean and standard errors of the mean within each group, respectively. **D** As in **C**, but for the GGMP cohort. **E** As in **C**, but for the CHNS cohort. **F** Relationship between the first two principal components of the microbial variation and type 2 diabetes. Logistic regression was used to examine the association of principal components (per SD unit) with type 2 diabetes, adjusted for age, sex, and BMI

We found that participants who were exposed to the Great Chinese Famine during the first 1000 days of life (born in 1959), rather than other periods, had a consistently lower observed OTUs in the GNHS (*P* = 0.0068), GGMP (*P* = 0.035), and CHNS cohorts (*P* = 0.018), compared with the no-exposed control group (born between 1962 and 1964) (Fig. 2A, B and Additional file 1: Table S4-S6). Similar trends were observed for the other alpha diversity indices (Additional file 1: Fig. S2 A-C and Additional file 1: Table S4-S6). Gut microbial alpha diversity was inversely associated with risk of type 2 diabetes in the three cohorts (Fig. 2B, pooled odds ratio 0.79, 95% CI 0.74, 0.85). This correlation remained consistent even after additional adjustments for dietary and lifestyle factors (Additional file 1: Fig. S3).

In the sensitivity analyses exploring the association of famine exposure with microbial alpha diversity, we obtained consistent results by adding a potential technique confounder (microbiome sequencing depth), dietary and lifestyle factors into the covariate list (Additional file 1: Table S7-S9), excluding the participants with type 2 diabetes (Additional file 1: Table S10-S12) or defining the participants born after 1978 as a new reference group (Additional file 1: Table S13-S14). The results were a bit weaker when we combined the above long exposure group (born in 1959) and those having a shorter exposure group (born during 1960–1961) (Additional file 1: Fig. S4 and Additional file 1: Table S15).

Given that age imbalance between the first 1000 days famine exposure group and the control group may confound the famine-gut microbiome analysis, similarly to a previous study [30], we set a new reference group by combining the non-exposed control group with the infancy and toddler exposed group to further control the influence of age. In the GNHS cohort, participants in the first 1000 days famine exposure group are on average 1.8 years older than the new reference group (Additional file 1: Fig. S5A). In the GGMP and CHNS cohorts, participants in the first 1000 days famine exposure group are on average 1.3 years (Additional file 1: Fig. S5B) and 1.2 years (Additional file 1: Fig. S5C) younger than the new reference group, respectively. Compared with the new reference group, participants who were exposed to the famine during the first 1000 days of life, rather than other periods, had consistently lower levels of observed OTUs in the three cohorts (Additional file 1: Fig. S5D and Additional file 1: Table S16). Similar trends were observed for other alpha diversity indices (Additional file 1: Table S16).

### Exposure to the Great Chinese Famine during the first 1000 days of life is associated with the alterations of the gut microbial landscape

PERMANOVA analysis showed that the gut microbial composition was significantly different between the first 1000 days famine exposure group and the no-exposed control group in the GNHS (*P* = 0.025, PERMANOVA test with 999 permutations) and CHNS (*P* = 0.009) cohorts. After performing principal coordinate analysis to reduce the dimension of the microbial data (Fig. 2C–E), we found that the alteration in microbial composition was only present in the first 1000 days famine exposure group when compared to the control group (Additional file 1: Fig. S6). Notably, the first 1000 days famine-induced gut microbial variation was mainly located in the first principal component (PCOA1, explained 17.4% of the total microbial variability) in the GNHS (*P* = 0.044, Additional file 1: Fig. S6 and Additional file 1: Table S17) and the second principal component (PCOA2, explained 11.9% of the total microbial variability) in the CHNS (*P* = 0.00035, Additional file 1: Fig. S6 and Additional file 1: Table S17). In the GGMP (Additional file 1: Fig. S6 and Additional file 1: Table S17), the variation of gut microbial composition between the first 1000 days famine exposure group and the no-exposed control group was mainly in the PCOA2 (*P* = 0.056, explained 11.9% of the total gut microbial variation). Further comparisons involving other groups are depicted in Additional file 1: Fig. S6 and Additional file 1: Table S17.

The compositional variation of the famine-disrupted gut microbiome was significantly associated with type 2 diabetes. In the GGMP and CHNS cohorts, the level of PCOA2 was lower in the first 1000 days famine exposure group than the no-exposed control group and was inversely associated with type 2 diabetes (Fig. 2F).

### Keystone taxa as drivers of gut microbial structure and diversity

**Fig. 3:**
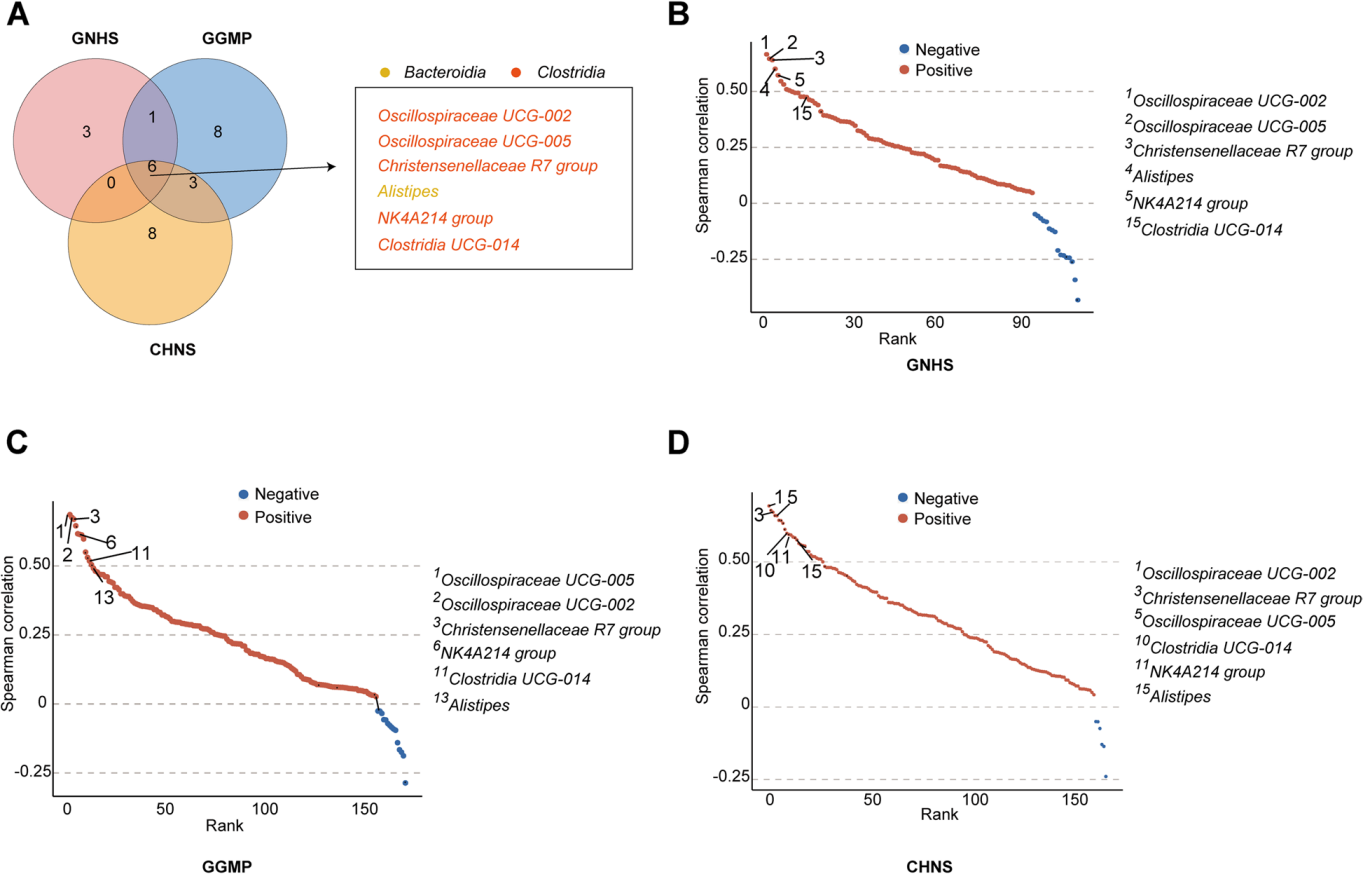
Keystone taxa as drivers of gut microbial diversity. **A** Venn diagram of the numbers and list of keystone taxa detected in each cohort. **B** Correlations between the gut microbial genera and observed OTUs in the GNHS. The values on the *x*-axis and *y*-axis represent the rank and Spearman’s coefficient of each genus’s correlation with observed OTUs, respectively. Only significant results were plotted in the figure, and the numbered genera represent the keystone taxa that were consistently detected across the three cohorts and their ranked numbers among all tested genera. **C** As in **B**, but for the GGMP cohort. **D** As in **B**, but for the CHNS cohort

Considering that alterations in microbial diversity and overall composition were consistently observed only in the first 1000 days famine-exposed groups across the three cohorts, we then delved into evaluating the potential influence of early-life famine exposure during this critical period on adult gut microbial keystone taxa. We firstly established microbial co-abundance networks by combining the Pearson, SparCC [31], and SPIEC-EASI [32] methods. We identified 10, 18, and 17 keystone taxa in the GNHS (Additional file 1: Fig. S7), GGMP (Additional file 1: Fig. S8), and CHNS cohorts (Additional file 1: Fig. S9), respectively. Notably, six genera (*Oscillospiraceae UCG-002*, *Oscillospiraceae UCG-005*, *Alistipes*, *NK4A214 group*, *Clostridia UCG 014*, and *Christensenellaceae R7 group*) were consistently identified as the shared keystone taxa across the three cohorts (Fig. 3A). The relative abundance of these taxa is relatively modest compared with other major gut microbial taxa (Additional file 1: Fig. S10). These keystone microbes are directly or indirectly connected with other gut microbes, playing a central position in the microbial ecosystem.

The identified shared keystone taxa were strongly associated with gut microbial alpha diversity (Fig. 3B–D). For example, the correlation coefficient between *Oscillospiraceae UCG-002* and observed OTUs was ranked highest in both the GNHS (Spearman’s *r* = 0.67, *P* < 0.001) and CHNS (Spearman’s *r* = 0.69, *P* < 0.001) cohorts and second highest in the GGMP cohort (Spearman’s *r* = 0.68, *P* < 0.001).

### Famine exposure during the first 1000 days of life, disruption of gut microbial keystone taxa, and type 2 diabetes

**Fig. 4:**
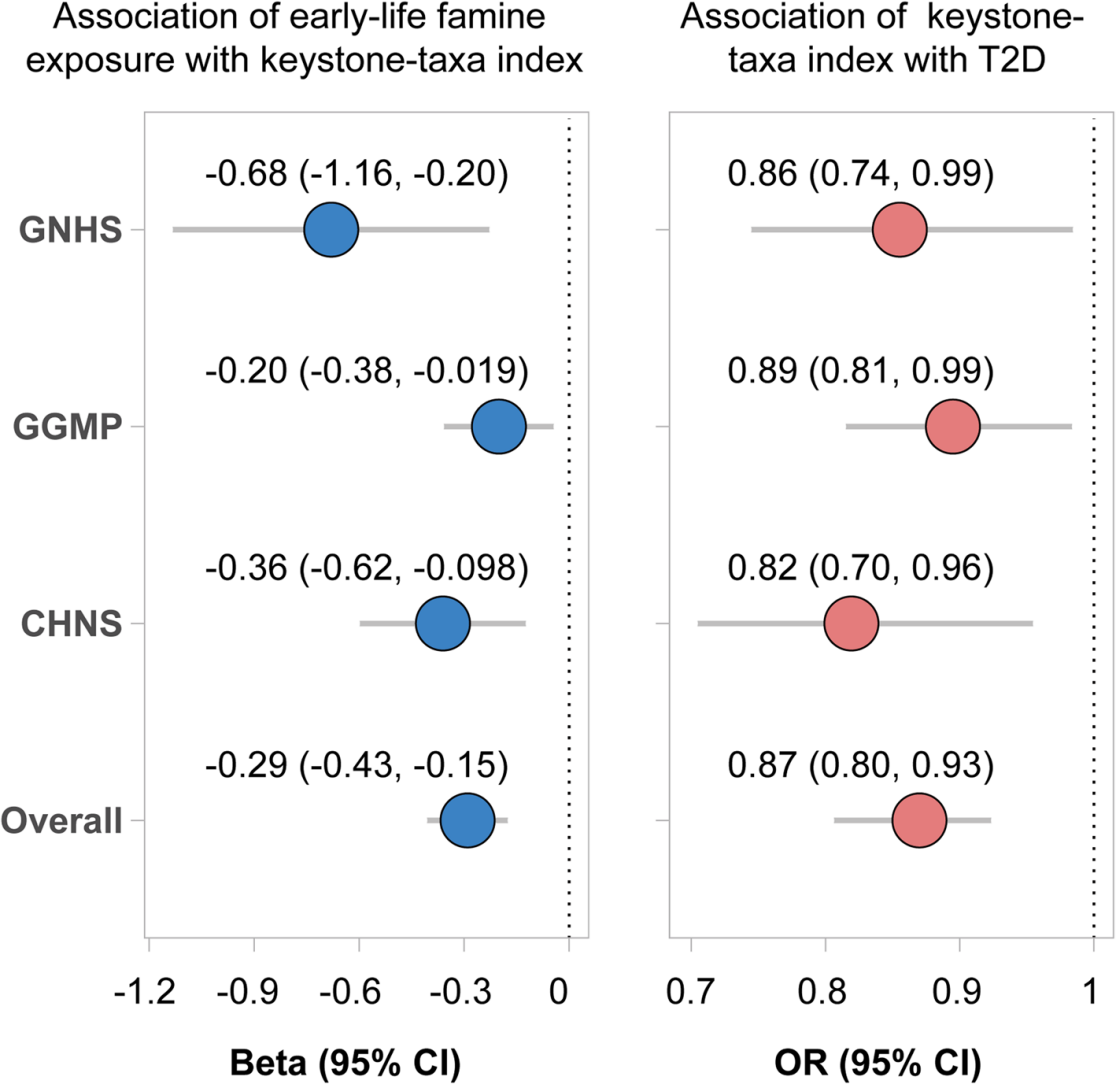
Famine exposure during the first 1000 days of life, disruption of keystone taxa, and type 2 diabetes. Left: association of early-life famine exposure with keystone taxa index. Keystone taxa index was constructed with the total abundance of the keystone taxa that were consistently detected in the three cohorts. Linear regression was used to estimate the difference in keystone taxa index between other groups and reference group, with adjustment of age, sex, BMI, and the use of hypoglycemic and hypolipidemic medications (yes/no for each). Here, we highlighted the comparation between the first 1000 days famine exposure and the control group in the three cohorts. Other results were present in the supplemental materials. Right: the associations of keystone taxa index (per SD unit) with type 2 diabetes using logistic regression in the three cohorts, adjusted for age, sex, and BMI. We combined the effect estimates from the three cohorts using random-effects meta-analysis

Overall, compared with the no-exposed control group, participants who were exposed to famine during the first 1000 days of life were associated with consistently lower levels of keystone taxa index across the three cohorts (Fig. 4, pooled beta − 0.29, 95% CI − 0.43, − 0.15). The taxa index was inversely associated with type 2 diabetes across the three cohorts (Fig. 4, per SD change, pooled odds ratio 0.87, 95% CI 0.80, 0.93).

Four keystone taxa (*Alistipes*, *Christensenellaceae R7 group*, *Oscillospiraceae UCG-002*, and *Oscillospiraceae UCG-005*), which were consistently detected in the three cohorts, were inversely associated with type 2 diabetes after meta-analyses (Additional file 1: Fig. S11). Importantly, the abundance of *Alistipes* and *Christensenellaceae R7 group* was lower in the first 1000 days famine exposure group than the no-exposed control group after conducting meta-analysis (Additional file 1: Fig. S12).

## Discussion

Accumulating epidemiological evidence suggests a link between early-life exposure to famine and increasing burden of chronic diseases, while little is known about whether famine exposure has a long-term impact on gut microbial health. In this cohort study, we reveal that participants exposed to the Great Chinese Famine during the first 1000 days of life are associated with the alterations of gut microbial diversity, composition, and keystone taxa. Importantly, the famine-induced disruptions in the gut microbiome are positively associated with the risk of type 2 diabetes.

Early life, especially the first 1000 days of life, is a critical window for the establishment and development of gut microbiota [14]. It is generally recognized that the initial microbes in infants are transmitted from the mother at delivery, but recent studies suggested that the exposure of the human to microbiota begins in utero [33]. After birth, the gut microbiome co-evolves with the host and starts stabilizing in later childhood [34, 35]. A previous study found that children with malnutrition, characterized by a relatively lower weight-for-length *Z* score, were associated with microbiome immaturity in early life [36]. A fecal microbiota transplantation study further demonstrated that undernutrition could causally perturbate the normal development of the gut microbiota, and the impaired growth phenotypes of undernourished donors could be transmitted to the recipient mice [37]. Our study further expands the existing knowledge by demonstrating that early-life malnutrition was closely associated with gut microbial dysbiosis in adults.

There is a crosstalk between the early-life gut microbiota and immune system, gastrointestinal integrity, and many other systems [38, 39]. The gut microbiota and its related metabolites may mediate the effects of environmental stress on human health and diseases in later life [40]. In our study, participants who were exposed to the Great Chinese Famine during the first 1000 days of life had a lower level of alpha diversity than the control participants. Gut microbial alpha diversity, a key quantity index of the overall microbial composition, was inversely associated with type 2 diabetes in the current study and prior report [41]. In addition, keystone taxa are directly or indirectly connected to other microbes and as drivers of microbial structure and functioning of microbial ecosystem [42]. In the current study, although with large variations in gut microbial composition across the geographic regions, six keystone taxa (*Oscillospiraceae UCG-002*, *Oscillospiraceae UCG-005*, *Alistipes*, *NK4A214 group*, *Clostridia UCG 014*, and *Christensenellaceae R7 group*) were consistently detected in the three cohorts. Importantly, *Christensenellaceae R7 group* and *Oscillospiraceae UCG-002* were also identified as the keystone taxa in the previous study [43] and were decreased in the first 1000 days famine exposed participants. *Christensenellaceae R7 group* was inversely associated with various circulating lipids and insulin resistance [41, 44, 45]. A higher abundance of *Oscillospiraceae UCG-002* could benefit insulin resistance and depressive symptoms [46]. In agreement with these results, the abundance of *Christensenellaceae R7 group* and *Oscillospiraceae UCG-002* were decreased in type 2 diabetes participants in the present study. *Alistipes* is one of the major butyrate-producing taxa and was mainly detected in the healthy human gastrointestinal tract [47, 48]. *Alistipes* had protective effects against dysglycemia and cardiovascular disease [47, 48] and are the key determinants of post-antibiotic ecological recovery in the gut [49]. In our study, *Alistipes* was enriched in the no-exposed control participants, and a higher abundance of *Alistipes* was associated with a lower risk of type 2 diabetes.

This study has several strengths. Firstly, this is the first study to comprehensively examine the lifelong impact of famine exposure during early life on the adult gut microbial ecosystem (microbial diversity, constitution, and keystone taxa) and type 2 diabetes. In addition, although the distinct study designs, variations in sample size, sequencing methods, and differences in population characteristics across these cohorts could potentially influence the robustness of effect estimates, the associations of early-life famine exposure with gut microbiome, and famine-induced disturbance in gut microbiome with type 2 diabetes, have been successfully established across all three independent cohorts, which supports the generalizability of the present findings. Our findings support the gut microbiome may play an important role in the DOHaD hypothesis. Finally, we identified several keystone taxa that were independent of the geographic regions. The identified keystone taxa may serve as the intervention targets for regulating the microbial ecosystem under the abnormal status.

This study has limitations. First, the absence of detailed individual-level data on famine exposure and its variations in severity between individuals may introduce some bias into the effect estimates. However, it is worth noting that during the famine period, food distribution primarily occurred through communal kitchens, effectively impacting the majority of participants living in the community [50]. Second, the famine during 1959–1961 affected all provinces of China; it is impossible to find no-exposed controls with a completely matched age structure as the famine births. Nevertheless, we used an age-balanced strategy to adjust age-related biases and obtained consistent findings across the three cohorts. Third, although we have adjusted for numerous covariates, the possibility of residual confounding could not be fully excluded due to the observational nature of the current study. Fourth, the sample size for the in utero exposure groups was relatively small due to the famine exposure. Fifth, because of the use of 16S rRNA data, associations of functional profiles of the gut microbiome could not be explored. Future omics data such as metagenomics and short-chain fatty acids may overcome these limitations. Finally, our findings on the associations of famine-induced disruption in the gut microbiome with type 2 diabetes are observational without clear causal evidence at this stage. Further longitudinal and interventional studies are needed to elucidate the underlying mechanism and potential causal direction.

## Conclusions

In conclusion, our results highlight the potential role of the gut microbiome in the DOHaD hypothesis, which contributes to our understanding of disease etiology and pathogenesis. The identified keystone taxa may serve as new interventions or therapeutic targets for type 2 diabetes. More investigations are needed to further replicate our present findings and reveal the detailed mechanism.

### Supplementary Information

**Additional file 1:** Supplementary methods. **Fig. S1. **Flow diagram of participants selection process. **Fig. S2. **Associations of famine exposure with gut microbial diversity. **Fig. S3. **Association of Observed OTUs with type 2 diabetes. **Fig. S4. **Association of in utero famine exposed with gut microbial diversity in the three cohorts. **Fig. S5. **Association of famine exposure with gut microbial diversity after age balance. **Fig. S6. **Shifts in the principal components of the microbial variation relative to the no-exposed control group. **Fig. S7.** Co-abundance network construction and keystone taxa identification in the GNHS cohort. **Fig. S8. **Co-abundance network construction and keystone taxa identification in the GGMP cohort. **Fig. S9. **Co-abundance network construction and keystone taxa identification in the CHNS cohort. **Fig. S10. **Mean relative abundance of keystone taxa in the three cohorts. **Fig. S11. **Association of keystone taxa with type 2 diabetes. **Fig. S12. **Association of early-life famine exposure with keystone taxa. **Table S1. **Characteristics of the participants included in the GNHS cohort. **Table S2. **Characteristics of the participants included in the GGMP cohort. **Table S3. **Characteristics of the participants included in the CHNS cohort. **Table S4. **Association of famine exposure with gut microbial diversity in the GNHS cohort (Model 1).** Table S5. **Association of famine exposure with gut microbial diversity in the GGMP cohort (Model 1).** Table S6.** Association of famine exposure with gut microbial diversity in the CHNS cohort (Model 1). **Table S7. **Association of famine exposure with gut microbial diversity in the GNHS cohort (Model 2). **Table S8. **Association of famine exposure with gut microbial diversity in the GGMP cohort (Model 2). **Table S9. **Association of famine exposure with gut microbial diversity in the CHNS cohort (Model 2). **Table S10. **Association of famine exposure with gut microbial diversity in the GNHS cohort (Model 3). **Table S11. **Association of famine exposure with gut microbial diversity in the GGMP cohort (Model 3). **Table S12. **Association of famine exposure with gut microbial diversity in the CHNS cohort (Model 3). **Table S13. **Association of famine exposure with gut microbial diversity in the GGMP cohort (Model 4). **Table S14. **Association of famine exposure with gut microbial diversity in the CHNS cohort (Model 4). **Table S15. **Association of in utero famine exposed with gut microbial diversity in the three cohorts. **Table S16. **Association of famine exposure with gut microbial diversity after age balance. **Table S17. **Shifts in the gut microbial composition relative to the no-exposed control group in the three cohorts.

## Data Availability

16S rRNA gene sequencing data of the Guangzhou Nutrition and Health Study (GNHS) are available in the Genome Sequence Archive (GSA) (https://ngdc.cncb.ac.cn/gsa/) at accession number CRA006769. 16S rRNA gene sequencing data of the Guangdong Gut Microbiome Project (GGMP) are available from the European Nucleotide Archive (https://www.ebi.ac.uk/ena/) at accession number PRJEB18535. The metadata of the GGMP are available in a previous publication (https://pubmed.ncbi.nlm.nih.gov/30250144/).

## Abbreviations

CHNS: China Health and Nutrition Survey
CLR: Centered log-ratio
DOHaD: Developmental origins of health and disease
GGMP: Guangdong Gut Microbiome Project
GNHS: Guangzhou Nutrition and Health Study
HbA1c: Glycated hemoglobin
PCOA: Principal coordinate analysis
