# Supplementary material for: Early-life exposure to the Great Chinese Famine and gut microbiome disruption across adulthood for type 2 diabetes: three population-based cohort studies
Source: BMC Med. 2023 Nov 1;21:414. doi: 10.1186/s12916-023-03123-y (PMC10619253; doi:10.1186/s12916-023-03123-y)
Supplement: Supplementary file 1 — Additional file 1: Supplementary methods. Fig. S1. Flow diagram of participants selection process. Fig. S2. Associations of famine exposure with gut microbial diversity. Fig. S3. Association of Observed OTUs with type 2 diabetes. Fig. S4. Association of in utero famine exposed with gut microbial diversity in the three cohorts. Fig. S5. Association of famine exposure with gut microbial diversity after age balance. Fig. S6. Shifts in the principal components of the microbial variation relative to the no-exposed control group. Fig. S7. Co-abundance network construction and keystone taxa identification in the GNHS cohort. Fig. S8. Co-abundance network construction and keystone taxa identification in the GGMP cohort. Fig. S9. Co-abundance network construction and keystone taxa identification in the CHNS cohort. Fig. S10. Mean relative abundance of keystone taxa in the three cohorts. Fig. S11. Association of keystone taxa with type 2 diabetes. Fig. S12. Association of early-life famine exposure with keystone taxa. Table S1. Characteristics of the participants included in the GNHS cohort. Table S2. Characteristics of the participants included in the GGMP cohort. Table S3. Characteristics of the participants included in the CHNS cohort. Table S4. Association of famine exposure with gut microbial diversity in the GNHS cohort (Model 1). Table S5. Association of famine exposure with gut microbial diversity in the GGMP cohort (Model 1). Table S6. Association of famine exposure with gut microbial diversity in the CHNS cohort (Model 1). Table S7. Association of famine exposure with gut microbial diversity in the GNHS cohort (Model 2). Table S8. Association of famine exposure with gut microbial diversity in the GGMP cohort (Model 2). Table S9. Association of famine exposure with gut microbial diversity in the CHNS cohort (Model 2). Table S10. Association of famine exposure with gut microbial diversity in the GNHS cohort (Model 3). Table S11. Association of famine exposure with gut [file 12916_2023_3123_MOESM1_ESM.docx]

**Additional file**

**Early-life exposure to the Great Chinese Famine and gut microbiome disruption across adulthood for** **type 2 diabetes: three population-based cohort studies**

**Supplementary methods**

*Gut microbiome analyses*

*GNHS cohort.* Stool samples were collected at a local study site within the School of Public Health at Sun Yat-sen University, and were transferred to a -80°C facility within 4 hours after collection. Total bacterial DNA was extracted by the QIAamp® DNA Stool Mini Kit (Qiagen, Hilden, Germany). The V3-V4 variable region of the 16S rRNA gene was amplified by the primers 341F (5’-CCTACGGGNGGCWGCAG-3’) and 805R (5’-GACTACHVGGGTATCTAATCC-3’). The V3-V4 region of the 16S rRNA gene was amplified and sequenced on an Illumina MiSeq platform. The raw sequencing reads were further processed with the Quantitative Insights Into Microbial Ecology 2 platform (QIIME 2) [1]. In summary, DADA2 [2] was used to filter sequencing reads with quality score Q<25 and to denoise reads into amplicon sequence variants (ASVs), resulting in feature tables and representative sequences. Taxonomy was assigned to ASVs using the naive Bayes taxonomy classifier against the Silva-138-99 reference sequences [3]. ASVs were rarefied at 5,000 reads to calculate alpha diversity. A total of 2 samples in the GNHS had fewer reads than the specified cut-off and hence were excluded from the analysis. The measures of alpha diversity at the ASV level include observed OTUs, Shannon’s diversity index, Faith’s phylogenetic diversity, and Pielou’s evenness.

*GGMP cohort*. Stool samples were collected by the participants themselves, who received detailed instructions about how to collect and store stool samples. The collected stool samples were immediately stored in participants’ refrigerators (–18 to –20°C) after collection. All stool samples were transported through a cold chain to the research laboratory (Guangdong CDC) within 3 days and stored in –20°C freezers until processing. Total bacterial DNA extraction was performed using a Fecal DNA Bead Isolation kit (Bioeasy, Shenzhen). The V4 region of the 16S rRNA gene was amplified by the primers 515F (5′-GTGCCAGCMGCCGCGGTAA-3′) and 806R (5’-GGACTACHVGGGTWTCTAAT-3′). V4 region of the 16S rRNA gene was amplified and sequenced on an Illumina Hiseq 2,500 platform. Sequencing data in the GGMP were processed using the same pipeline as described above for the GNHS cohort. For alpha diversity, reads were rarefied to an even depth of 5,000 reads. A total of 4 samples in the GGMP had fewer reads than the specified cut-off and hence were excluded from the analysis.

*CHNS cohort*. Stool samples were collected by the participants themselves, who received instructions for the collection and storage process, and were immediately frozen at −20°C refrigerators after collection. All stool samples were transported through a cold chain to the central laboratory within 1–2 days and stored at −20°C until processing. Bacterial DNA extraction was based on a bead-beating procedure with TIANGEN DNA extraction kits (TIANGEN Biotech, Beijing, China). The 16S rRNA V4 region was amplified by the primers 515F (5′-GTGCCAGCMGCCGCGGTAA-3′) and 806R (5’-GGACTACHVGGGTWTCTAAT-3′). V4 region of 16S rRNA gene was amplified and sequenced on Illumina HiSeq PE-250 platform. Sequencing data in the CHNS were processed using the same pipeline as described above for the GNHS cohort. For alpha diversity, reads were rarefied to an even depth of 5,000 reads. A total of 2 samples in the CHNS had fewer reads than the specified cut-off and hence were excluded from the analysis.

**References**

1. Bolyen E, Rideout JR, Dillon MR, Bokulich NA, Abnet CC, Al-Ghalith GA, et al. Reproducible, interactive, scalable and extensible microbiome data science using QIIME 2. Nature biotechnology. 2019;37: 852–7.

2. Callahan BJ, McMurdie PJ, Rosen MJ, Han AW, Johnson AJA, Holmes SP. DADA2: High-resolution sample inference from Illumina amplicon data. Nature methods. 2016;13(7):581–3.

3. Yilmaz P, Parfrey LW, Yarza P, Gerken J, Pruesse E, Quast C, et al. The SILVA and “All-species Living Tree Project (LTP)” taxonomic frameworks. Nucleic Acids Research. 2014;42(D1): D643–8.

**Fig.S1. Flow diagram of participants selection process.**


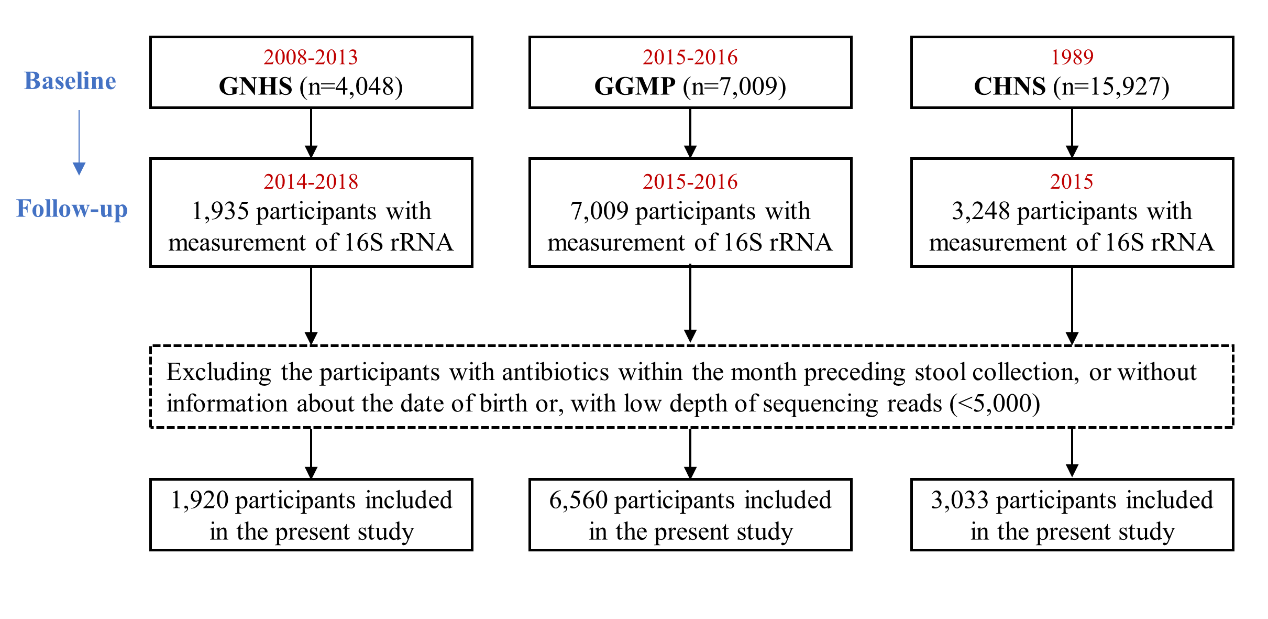


**Fig.S2. Associations of famine exposure with gut microbial diversity. A.** Left: Distribution of Shannon’s diversity across different groups. To be comparable, Shannon’s diversity was presented as the mean and 95% confidence interval of the z-scaled values. Here, participants in the no-exposed control group (NE2, born in 1962-1964) were considered as the reference group. Right: Shifts in Shannon’s diversity in no-exposed (NE1) and different famine exposed groups (E1-E8) compared to the unexposed control group (NE2). Linear regression was used to estimate the difference, with adjustment of age, sex, BMI, and the use of hypoglycemic and hypolipidemic medications (yes/no for each). **B.** As in A, but for Pielou’s evenness. **C.** As in A, but for Faith’s phylogenetic diversity.

**
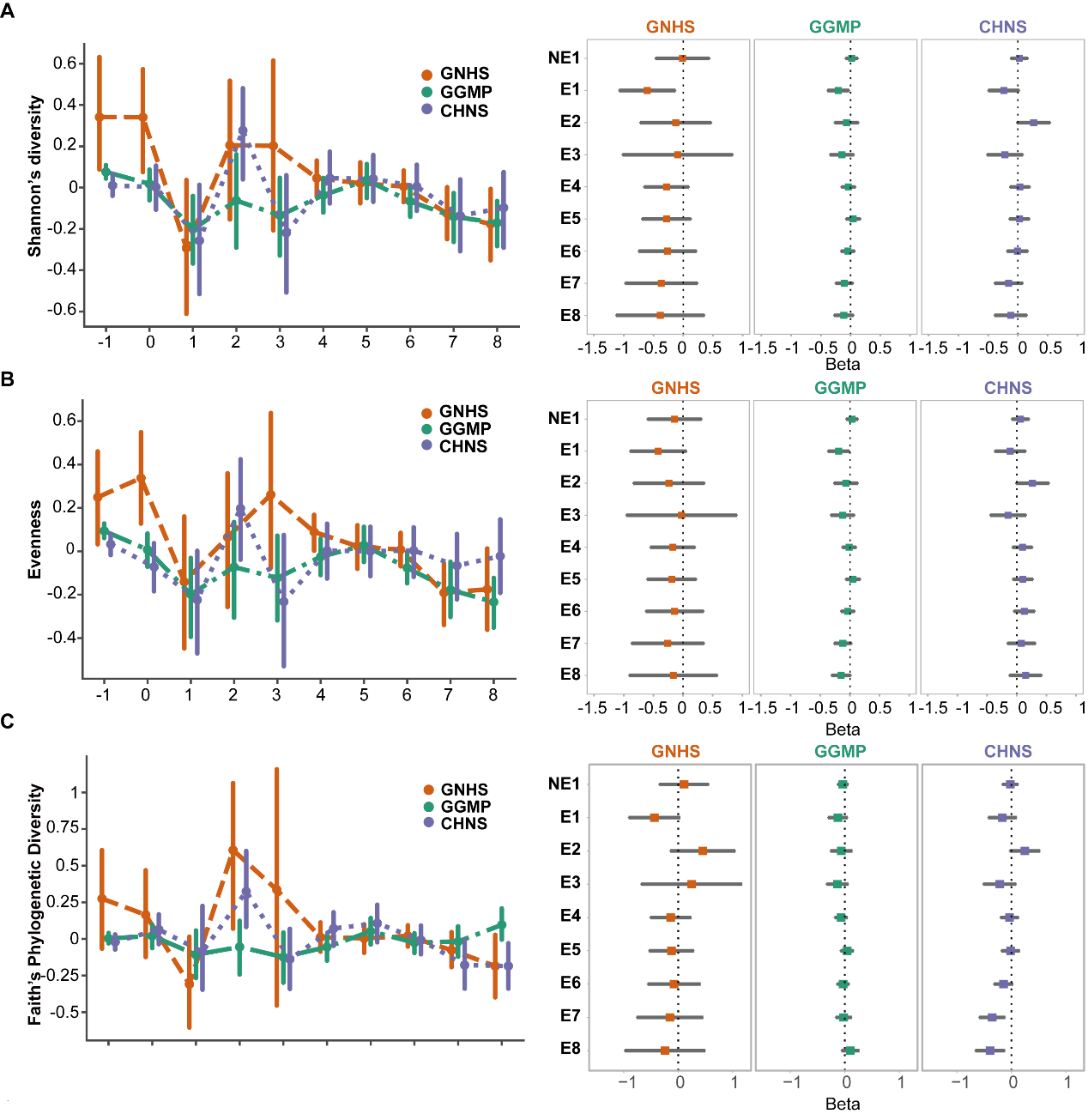
**

**Fig.S3. Association of Observed OTUs with type 2 diabetes.** Logistic regression was used to examine the association of Observed OTUs (per SD unit) with type 2 diabetes, adjusted for age, sex, BMI, dietary and lifestyle factors. We combined the effect estimates from the three cohorts using random-effects meta-analysis.**
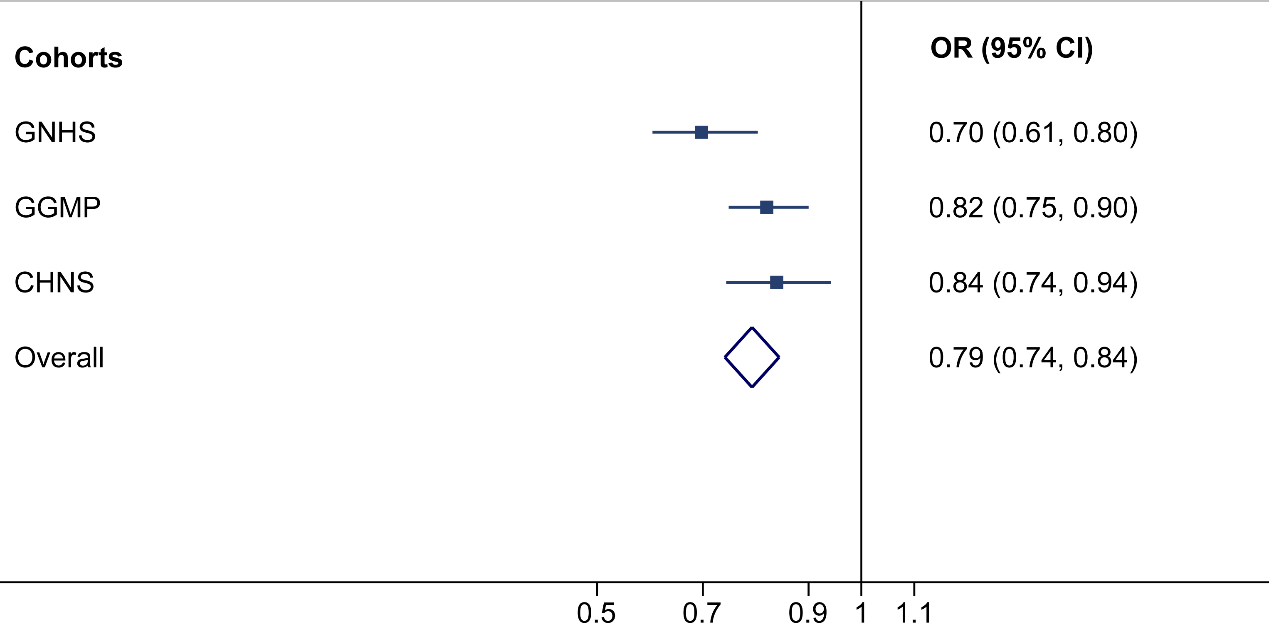
**

**Fig.S4. Association of in utero famine exposed with gut microbial diversity in the three cohorts.** Shifts in alpha diversity indexes in utero famine exposed group (born in 1959-1961) compared with the unexposed control group (born in 1962-1964). Linear regression was used to estimate the difference, with adjustment of age, sex, BMI, and the use of hypoglycemic and hypolipidemic medications (yes/no for each).

**
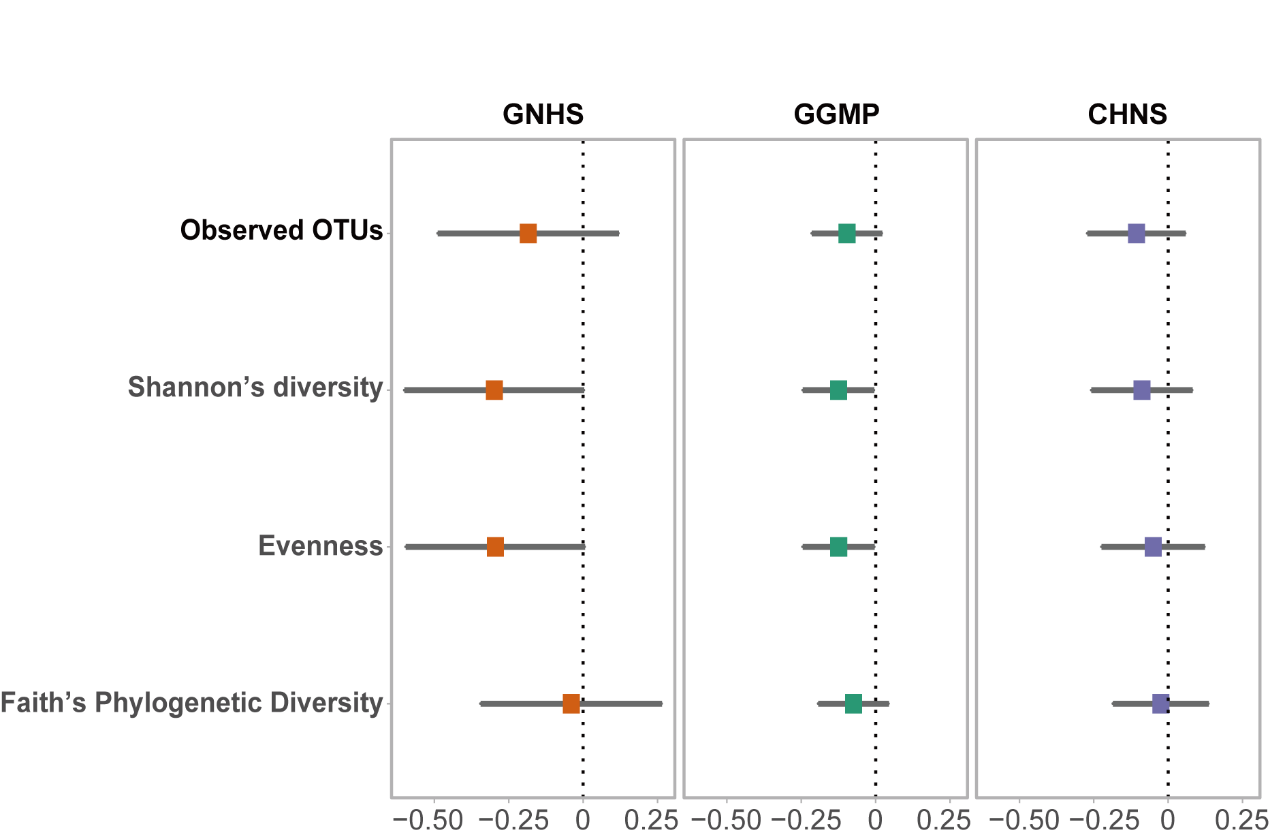
**

**Fig.S5. Association of famine exposure with gut microbial diversity after age balance. A-C.** Distribution of age in three in the utero exposed groups (E1-E3) and new-reference group in the GNHS, GGMP and CHNS cohorts, respectively. The new reference group represents the combination of participants born in 1962-1964 (NE2, no-exposed control group) and 1956-1958 (E4, infancy-and toddler-exposed group). **D.** Shifts in observed OTUs among different utero exposed groups (E1-E3), compared with the new-reference group. Linear regression was used to estimate the difference, with adjustment of age, sex, BMI, and the use of hypoglycemic and hypolipidemic medications (yes/no for each).

**
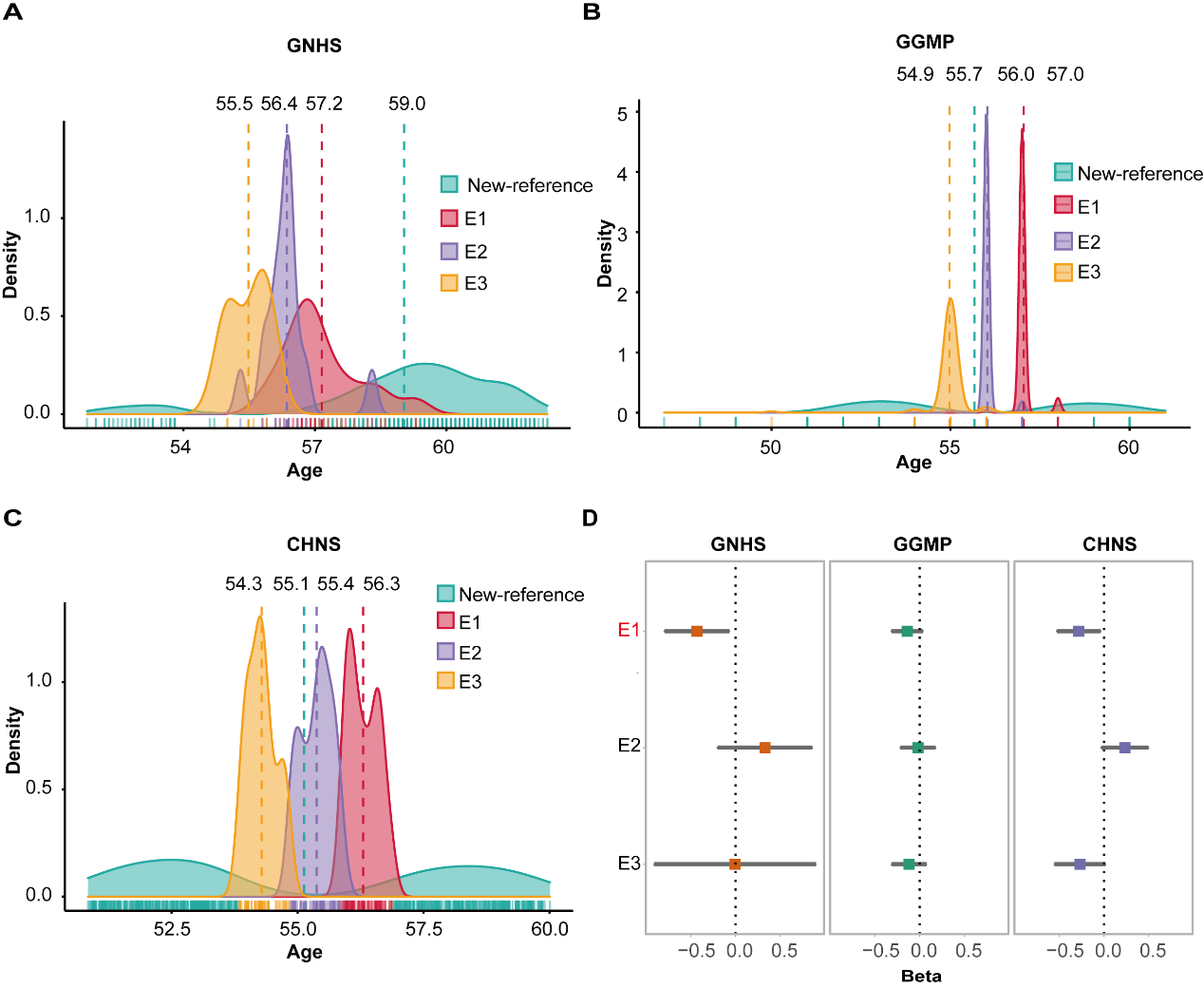
**

**Fig.S6. Shifts in the principal components of the microbial variation relative to the no-exposed control group.** Linear regression was used to estimate the difference between each tested group and the no-exposed control group, with adjustment of age, sex, BMI, and the use of hypoglycemic and hypolipidemic medications (yes/no for each).

**
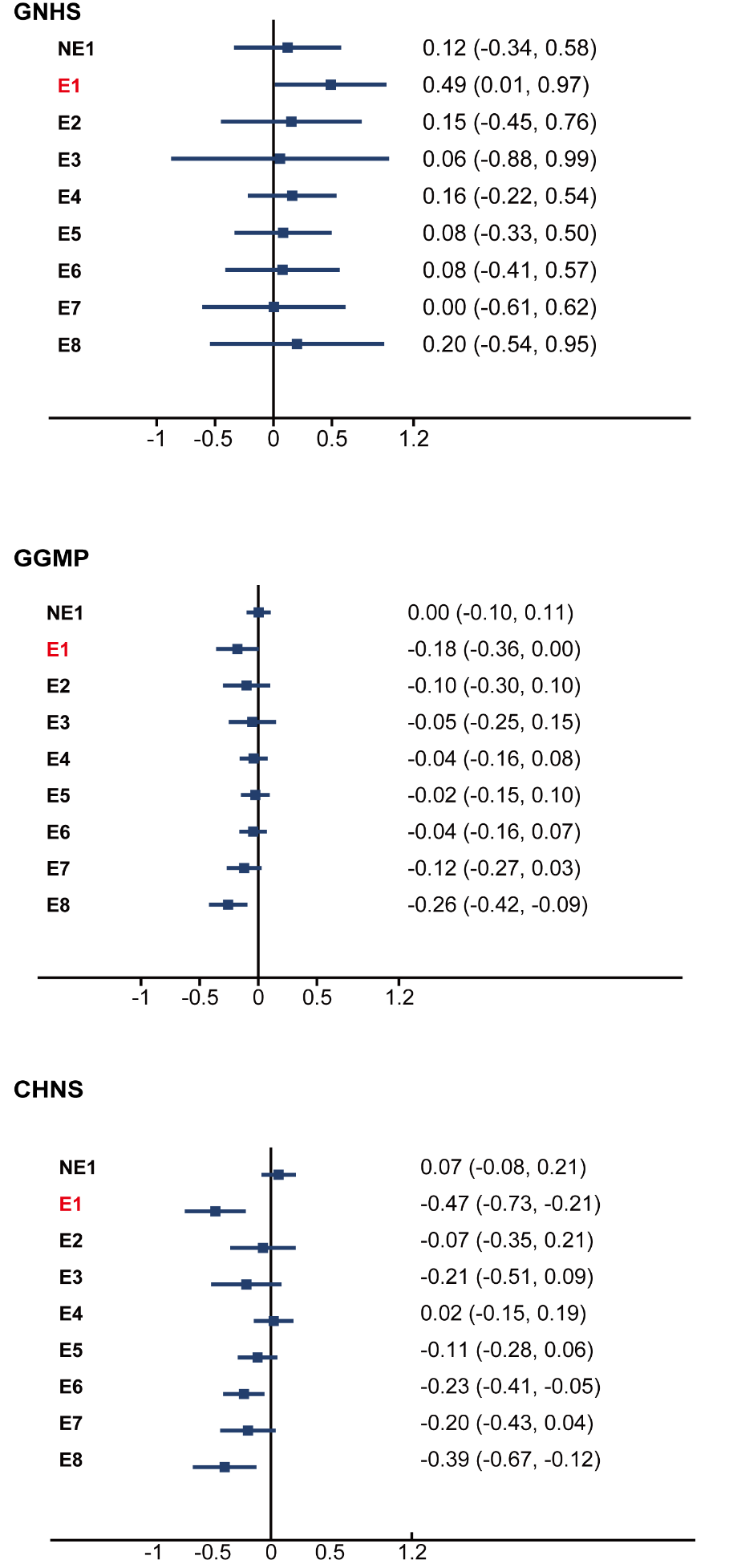
**

**Fig.S7. Co-abundance network construction and keystone taxa identification in the GNHS cohort. A.** Co-abundance network constructed by Pearson analysis. we used multiplicative imputation to handle zero values and used centered log-ratio (CLR) transformation to move compositional data from the simplex to real space. Correlations with FDR adjusted *P* values < 0.05 and with a magnitude above 0.3 were selected for further visualization and network analysis. Red edge, positive association; blue edge, negative association. **B.** Co-abundance network constructed by SparCC analysis. Correlations with FDR adjusted *P* values < 0.05 and with a magnitude above 0.2 were selected for further visualization and network analysis. **C.** Co-abundance network constructed by SPIEC-EASI analysis. The numbered taxa represent the keystone taxa identified by the eigenvector centrality. The clusters of nodes were marked with different colors. **D.** Venn diagram of the numbers of keystone taxa identified by each method. **E.** Supplemental information for the A-D.

**
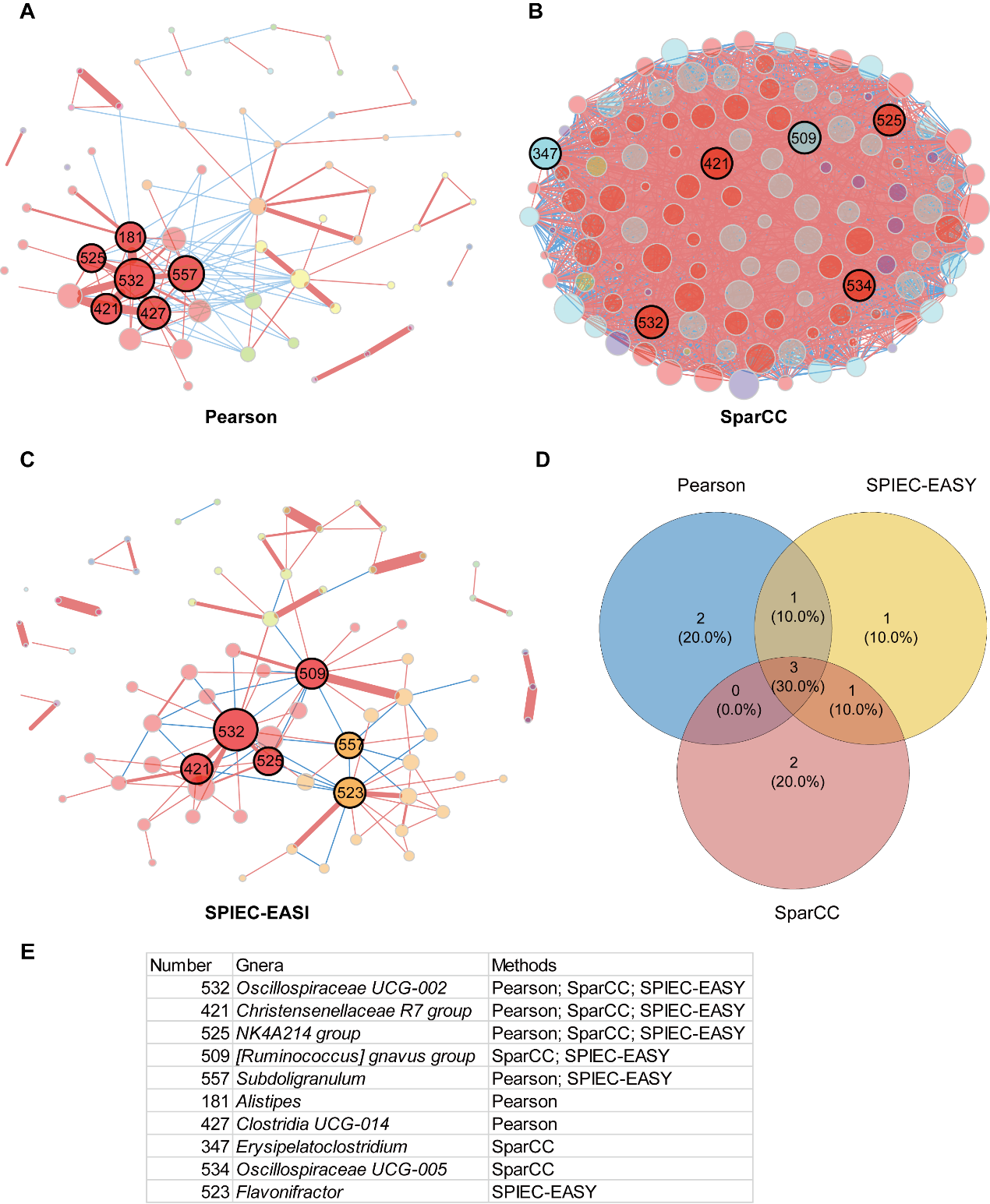
**

**Fig.S8. Co-abundance network construction and keystone taxa identification in the GGMP cohort.** A-E, see Fig.S6.

**
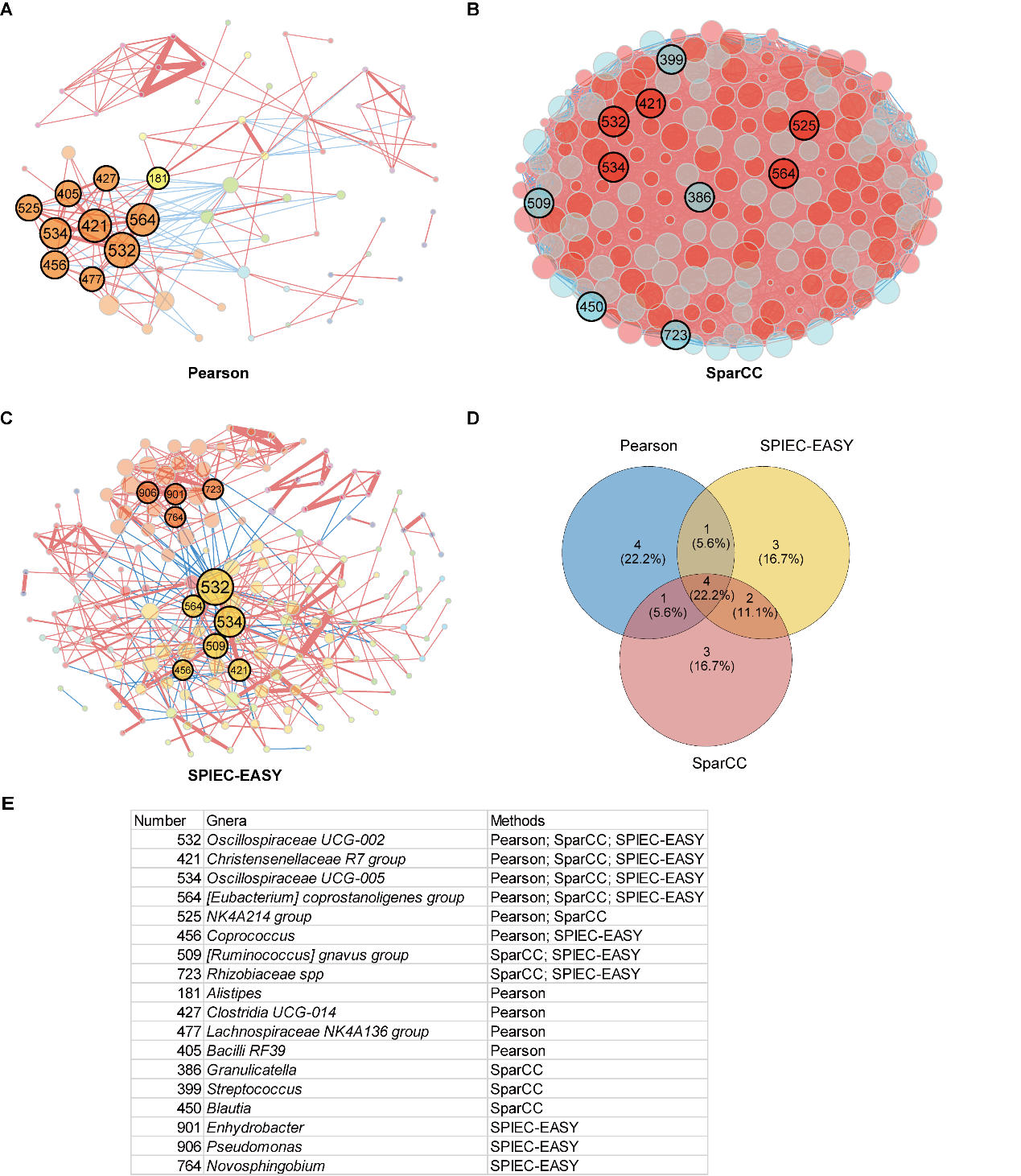
**

**Fig.S9. Co-abundance network construction and keystone taxa identification in the CHNS cohort.** A-E, see Fig.S6.**
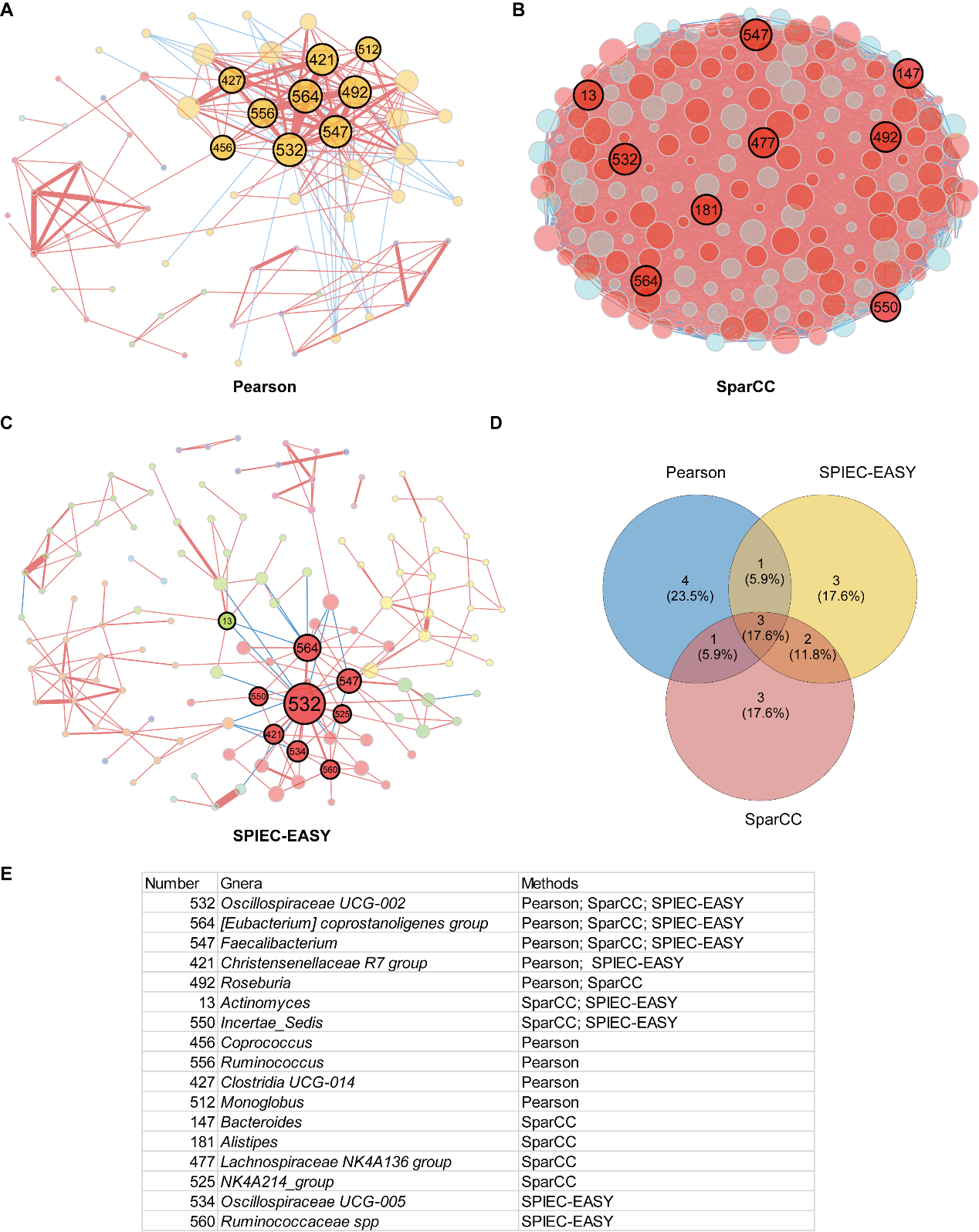
**

**Fig.S10. Mean relative abundance of keystone taxa in the three cohorts.**

**
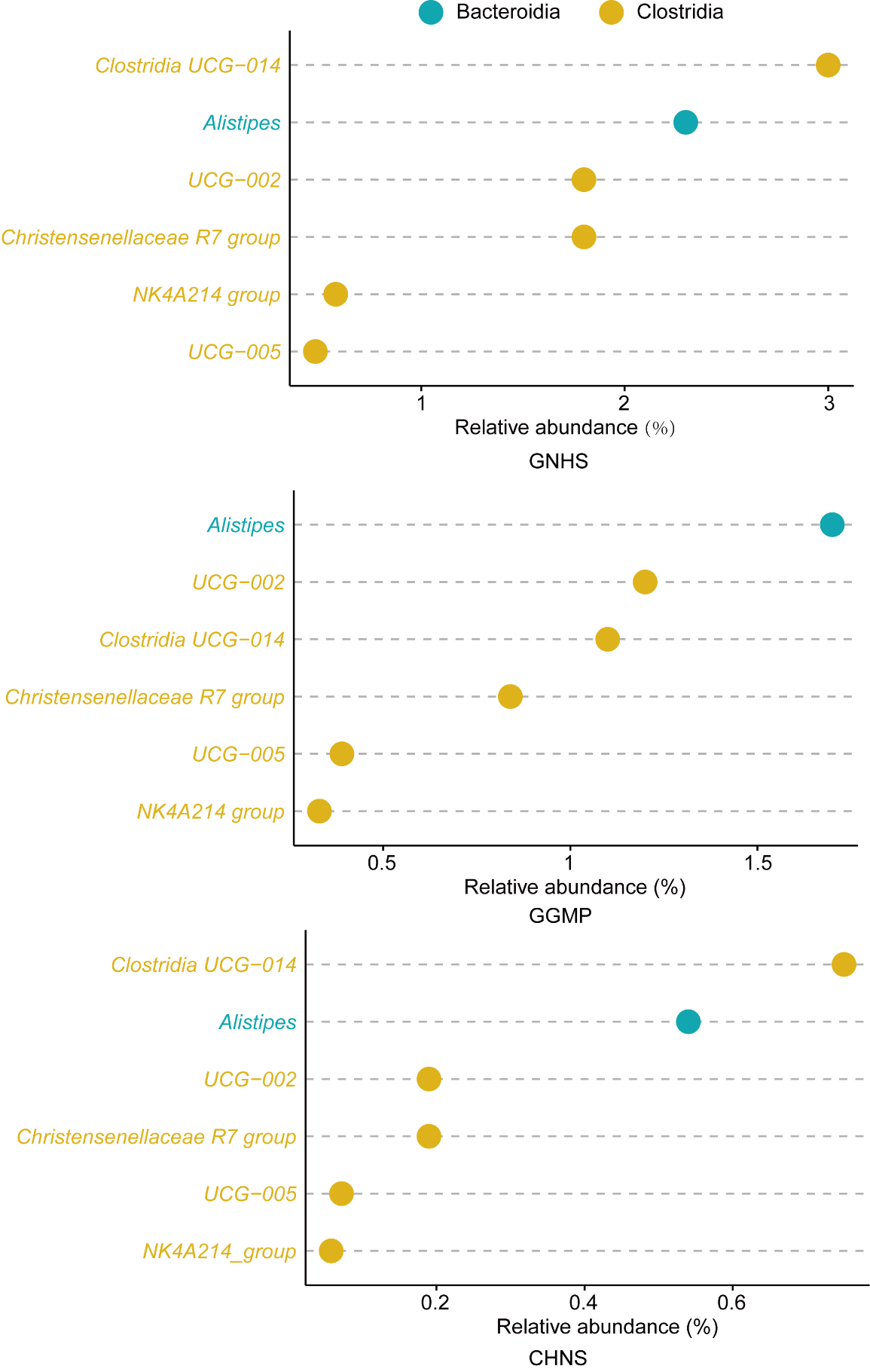
**

**Fig.S11. Association of keystone taxa with type 2 diabetes.** The associations of keystone taxa (per SD unit) with type 2 diabetes using logistic regression in the three cohorts, adjusted for age, sex, and BMI. We combined the effect estimates from the three cohorts using random-effects meta-analysis.

**
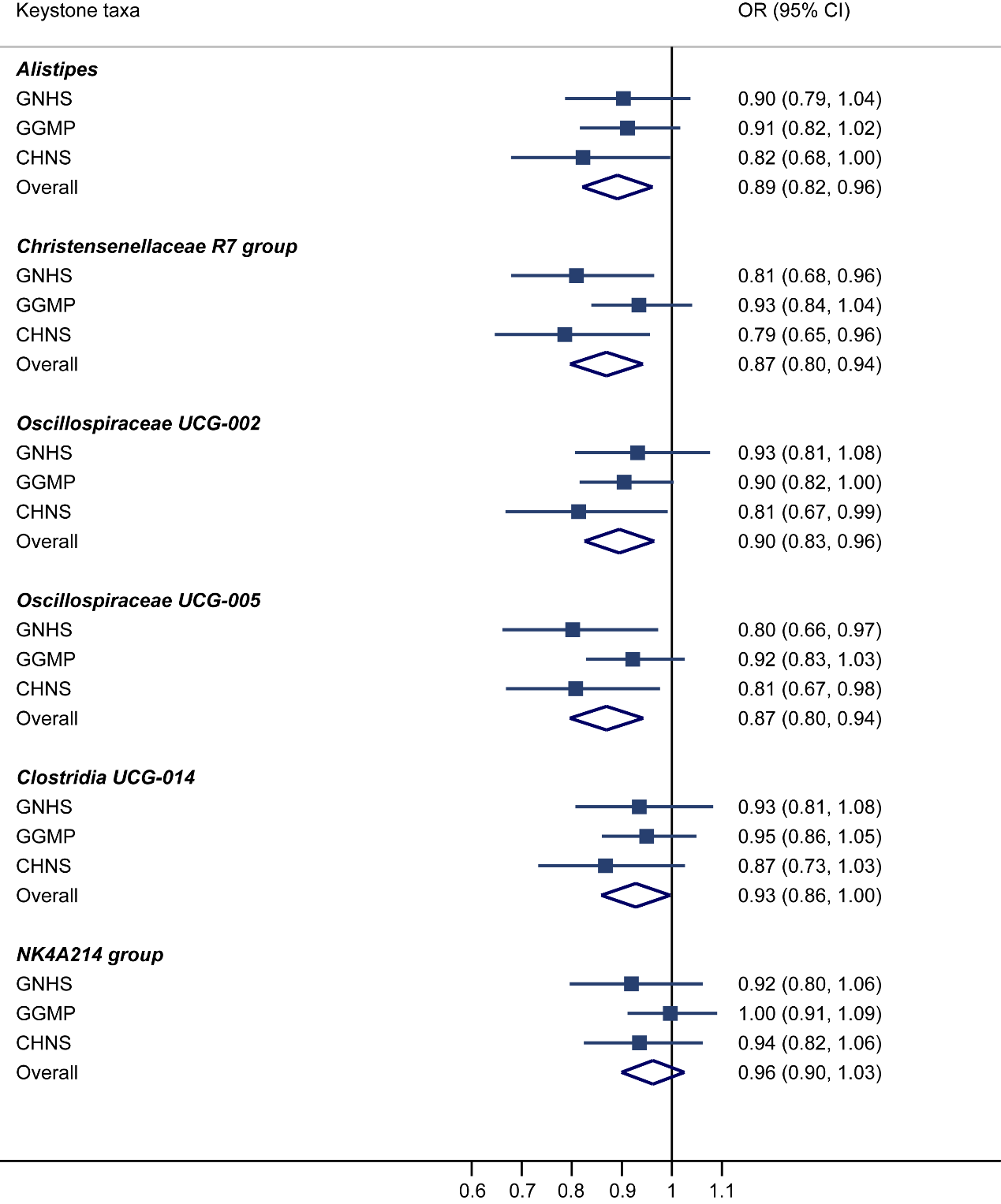
**

**Fig.S12. Association of early-life famine exposure with keystone taxa.** Linear regression was used to estimate the difference in keystone taxa between the first 1,000 days famine exposure and reference group, with adjustment of age, sex, BMI, and the use of hypoglycemic and hypolipidemic medications (yes/no for each). We combined the effect estimates from the three cohorts using random-effects meta-analysis.

**
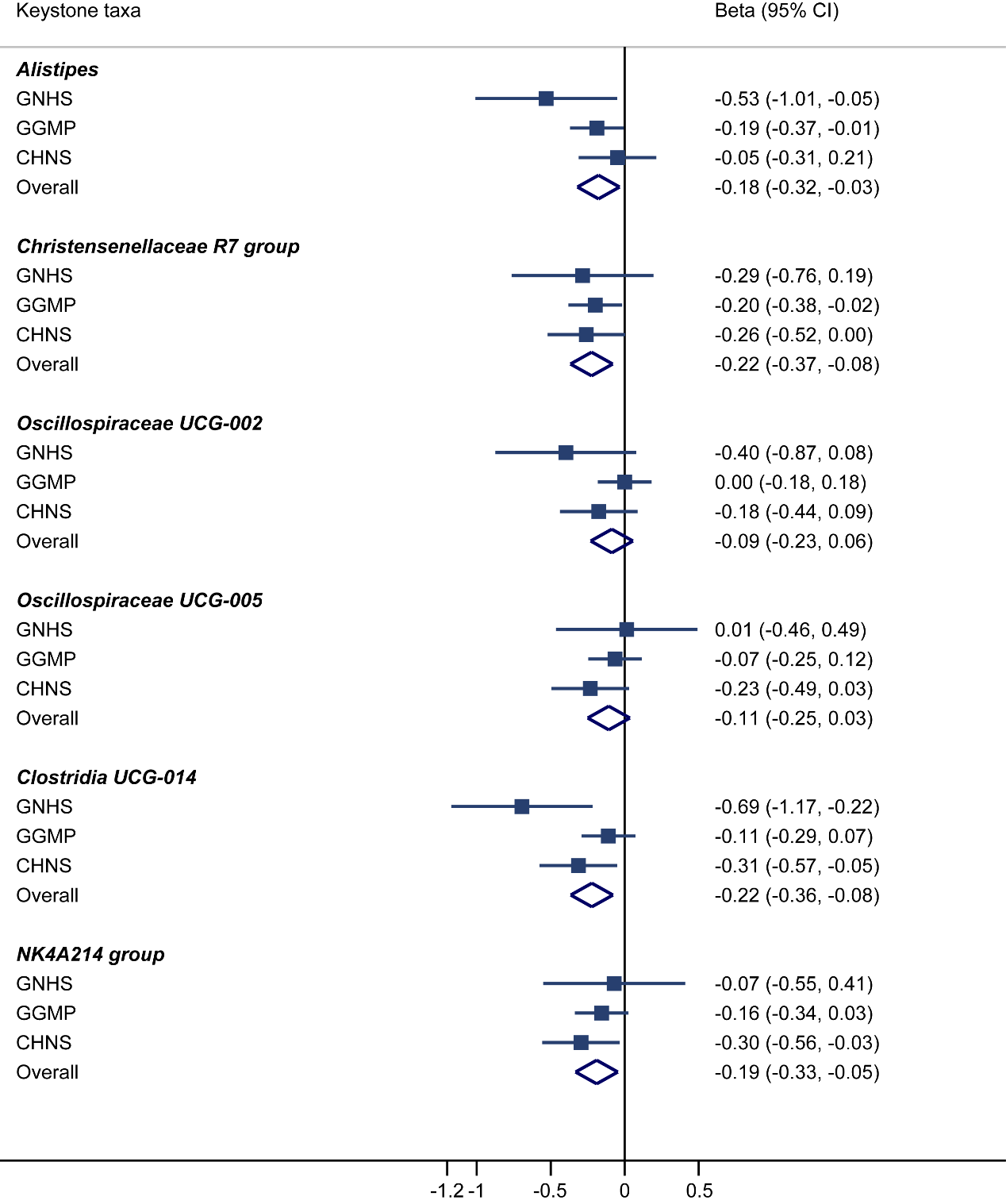
**

**Table S1. Characteristics of the participants included in the GNHS cohort.**

|  | **No-exposed control** | **Utero exposed group1 (born in 1959)** | **Utero exposed group2 (born in 1960)** | **Utero exposed group3 (born in 1961)** | **Infancy- and toddler-exposed** | **Preschooler exposed** | **School-aged child exposed** | **Adolescent exposed** | **Adult exposed** | **Other no-exposed participants** |
| --- | --- | --- | --- | --- | --- | --- | --- | --- | --- | --- |
| Number of participants | 37 | 34 | 15 | 5 | 341 | 425 | 670 | 261 | 91 | 41 |
| Age, years | 53.20 (0.95) | 57.15 (0.88) | 56.36 (0.65) | 55.48 (0.45) | 59.66 (1.27) | 62.43 (1.23) | 66.78 (2.04) | 72.38 (1.77) | 77.39 (2.17) | 48.62 (2.22) |
| Women, n (%) | 19 (51.4%) | 29 (85.3%) | 10 (66.7%) | 4 (80.0%) | 266 (78.0%) | 311 (73.2%) | 447 (66.7%) | 138 (52.9%) | 36 (39.6%) | 28 (68.3%) |
| BMI, kg/m^2^ | 23.53 (2.47) | 23.05 (1.99) | 23.74 (4.97) | 24.25 (1.64) | 23.58 (3.55) | 23.44 (3.17) | 23.75 (3.41) | 23.82 (3.46) | 23.16 (2.87) | 23.52 (2.83) |
| Type 2 diabetes, n (%) | 5 (13.5%) | 4 (11.8%) | 1 (6.7%) | 0 (0.0%) | 24 (7.0%) | 48 (11.3%) | 111 (16.6%) | 46 (17.6%) | 26 (28.6%) | 3 (7.3%) |

**Table S2. Characteristics of the participants included in the GGMP cohort.**

|  | **No-exposed control** | **Utero exposed group1 (born in 1959)** | **Utero exposed group2 (born in 1960)** | **Utero exposed group3 (born in 1961)** | **Infancy- and toddler-exposed** | **Preschooler exposed** | **School-aged child exposed** | **Adolescent exposed** | **Adult exposed** | **Other no-exposed participants** |
| --- | --- | --- | --- | --- | --- | --- | --- | --- | --- | --- |
| Number of participants | 622 | 144 | 111 | 111 | 512 | 502 | 822 | 380 | 430 | 2,926 |
| Age, years | 52.98 (0.97) | 57.04 (0.23) | 56.03 (0.21) | 54.97 (0.55) | 58.93 (0.91) | 61.93 (0.99) | 66.12 (1.69) | 71.76 (1.61) | 79.51 (3.72) | 39.27 (8.84) |
| Women, n (%) | 352 (56.6%) | 74 (51.4%) | 61 (55.0%) | 65 (58.6%) | 286 (55.9%) | 280 (55.8%) | 399 (48.5%) | 182 (47.9%) | 214 (49.8%) | 1701 (58.1%) |
| BMI, kg/m^2^ | 23.76 (3.29) | 23.94 (3.39) | 24.00 (3.73) | 23.97 (3.54) | 23.50 (3.28) | 23.43 (3.57) | 23.32 (3.39) | 22.94 (3.56) | 22.47 (3.53) | 23.38 (3.55) |
| Type 2 diabetes, n (%) | 67 (10.8%) | 15 (10.4%) | 14 (12.6%) | 14 (12.6%) | 58 (11.3%) | 58 (11.6%) | 101 (12.3%) | 52 (13.7%) | 47 (10.9%) | 127 (4.3%) |

**Table S3. Characteristics of the participants included in the CHNS cohort.**

|  | **No-exposed control** | **Utero exposed group1 (born in 1959)** | **Utero exposed group2 (born in 1960)** | **Utero exposed group3 (born in 1961)** | **Infancy- and toddler-exposed** | **Preschooler exposed** | **School-aged child exposed** | **Adolescent exposed** | **Adult exposed** | **Other no-exposed participants** |
| --- | --- | --- | --- | --- | --- | --- | --- | --- | --- | --- |
| Number of participants | 303 | 69 | 58 | 48 | 259 | 300 | 340 | 137 | 109 | 1410 |
| Age, years | 52.36 (0.85) | 56.30 (0.30) | 55.38 (0.31) | 54.29 (0.28) | 58.37 (0.89) | 61.34 (0.86) | 65.27 (1.72) | 71.13 (1.52) | 76.57 (7.02) | 40.44 (7.33) |
| Women, n (%) | 162 (53.5%) | 28 (40.6%) | 35 (60.3%) | 22 (45.8%) | 130 (50.2%) | 158 (52.7%) | 176 (51.8%) | 73 (53.3%) | 46 (42.2%) | 724 (51.3%) |
| BMI, kg/m^2^ | 24.50 (3.47) | 24.04 (3.49) | 25.78 (10.76) | 24.03 (2.77) | 24.02 (3.28) | 24.12 (3.57) | 24.46 (3.69) | 24.38 (3.73) | 23.85 (4.03) | 24.15 (3.63) |
| Type 2 diabetes, n (%) | 44 (14.6%) | 13 (18.8%) | 7 (12.1%) | 7 (14.6%) | 43 (16.6%) | 60 (20.1%) | 60 (17.8%) | 28 (20.4%) | 23 (21.3%) | 83 (5.9%) |

**Table S4. Association of famine exposure with gut microbial diversity in the GNHS cohort (Model 1)*.**

| **Outcome** | **Group** | **Adjusted beta** | **Lower confidence interval** | **Higher confidence interval** | **P value** |
| --- | --- | --- | --- | --- | --- |
| Observed OTUs | E1 | -0.65 | -1.13 | -0.18 | 0.0068 |
| Observed OTUs | E2 | 0.07 | -0.53 | 0.67 | 0.8263 |
| Observed OTUs | E3 | -0.20 | -1.13 | 0.72 | 0.6654 |
| Observed OTUs | E4 | -0.37 | -0.75 | 0.00 | 0.0527 |
| Observed OTUs | E5 | -0.38 | -0.79 | 0.03 | 0.0727 |
| Observed OTUs | E6 | -0.42 | -0.91 | 0.07 | 0.0898 |
| Observed OTUs | E7 | -0.47 | -1.14 | 0.08 | 0.0865 |
| Observed OTUs | E8 | -0.70 | -1.44 | 0.04 | 0.0649 |
| Observed OTUs | NE1 | 0.22 | -0.24 | 0.67 | 0.3453 |
| Shannon | E1 | -0.60 | -1.08 | -0.13 | 0.0124 |
| Shannon | E2 | -0.13 | -0.73 | 0.47 | 0.6790 |
| Shannon | E3 | -0.09 | -1.02 | 0.83 | 0.8425 |
| Shannon | E4 | -0.28 | -0.66 | 0.10 | 0.1435 |
| Shannon | E5 | -0.28 | -0.70 | 0.13 | 0.1812 |
| Shannon | E6 | -0.26 | -0.75 | 0.22 | 0.2868 |
| Shannon | E7 | -0.37 | -0.98 | 0.24 | 0.2368 |
| Shannon | E8 | -0.39 | -1.13 | 0.36 | 0.3088 |
| Shannon | NE1 | -0.01 | -0.47 | 0.44 | 0.9594 |
| Faith's Phylogenetic Diversity | E1 | -0.44 | -0.91 | 0.03 | 0.0693 |
| Faith's Phylogenetic Diversity | E2 | 0.45 | -0.15 | 1.05 | 0.1379 |
| Faith's Phylogenetic Diversity | E3 | 0.25 | -0.68 | 1.17 | 0.6032 |
| Faith's Phylogenetic Diversity | E4 | -0.14 | -0.52 | 0.24 | 0.4725 |
| Faith's Phylogenetic Diversity | E5 | -0.12 | -0.54 | 0.29 | 0.5576 |
| Faith's Phylogenetic Diversity | E6 | -0.08 | -0.56 | 0.41 | 0.7545 |
| Faith's Phylogenetic Diversity | E7 | -0.15 | -0.76 | 0.46 | 0.6242 |
| Faith's Phylogenetic Diversity | E8 | -0.24 | -0.98 | 0.50 | 0.5201 |
| Faith's Phylogenetic Diversity | NE1 | 0.11 | -0.35 | 0.56 | 0.6448 |
| Evenness | E1 | -0.42 | -0.89 | 0.06 | 0.0855 |
| Evenness | E2 | -0.24 | -0.84 | 0.36 | 0.4397 |
| Evenness | E3 | -0.03 | -0.96 | 0.91 | 0.9565 |
| Evenness | E4 | -0.17 | -0.55 | 0.21 | 0.3708 |
| Evenness | E5 | -0.19 | -0.60 | 0.23 | 0.3702 |
| Evenness | E6 | -0.14 | -0.63 | 0.35 | 0.5736 |
| Evenness | E7 | -0.26 | -0.87 | 0.36 | 0.4105 |
| Evenness | E8 | -0.16 | -0.91 | 0.58 | 0.6680 |
| Evenness | NE1 | -0.14 | -0.60 | 0.31 | 0.5381 |

* Model1:

Linear regression: Diversity index ~ β1Group + β2 age + β3 sex + β4 BMI + β5 use of hypoglycemic or hypolipidemic medications + ϵ. The no-exposed control group (NE2, born in 1962-1964) was used as the reference group. The comparable groups including the the no-exposed group (NE1, born after 1964), three in utero exposed groups (E1-E3, born in 1959, 1960, and 1961, respectively), infancy- and toddler-exposed group (E4, born in 1956-1958), preschooler exposed group (E5, born in 1953-1955), school-aged child exposed group (E6, born in 1947-1952), adolescent exposed group (E7, born in 1942-1946) and adult exposed group (E8, born before 1942).

**Table S5. Association of famine exposure with gut microbial diversity in the GGMP cohort (Model 1)*.**

| **Outcome** | **Group** | **Adjusted beta** | **Lower confidence interval** | **Higher confidence interval** | **P value** |
| --- | --- | --- | --- | --- | --- |
| Observed OTUs | E1 | -0.20 | -0.38 | -0.01 | 0.0345 |
| Observed OTUs | E2 | -0.07 | -0.28 | 0.13 | 0.4765 |
| Observed OTUs | E3 | -0.16 | -0.36 | 0.04 | 0.1210 |
| Observed OTUs | E4 | -0.10 | -0.22 | 0.02 | 0.1144 |
| Observed OTUs | E5 | 0.01 | -0.12 | 0.13 | 0.9225 |
| Observed OTUs | E6 | -0.06 | -0.18 | 0.06 | 0.3102 |
| Observed OTUs | E7 | -0.03 | -0.18 | 0.12 | 0.6610 |
| Observed OTUs | E8 | 0.03 | -0.13 | 0.19 | 0.7192 |
| Observed OTUs | NE1 | -0.05 | -0.15 | 0.05 | 0.3423 |
| Shannon | E1 | -0.21 | -0.39 | -0.03 | 0.0257 |
| Shannon | E2 | -0.07 | -0.27 | 0.13 | 0.5044 |
| Shannon | E3 | -0.14 | -0.35 | 0.06 | 0.1603 |
| Shannon | E4 | -0.04 | -0.16 | 0.08 | 0.5416 |
| Shannon | E5 | 0.04 | -0.08 | 0.17 | 0.4846 |
| Shannon | E6 | -0.05 | -0.16 | 0.07 | 0.4478 |
| Shannon | E7 | -0.11 | -0.25 | 0.04 | 0.1659 |
| Shannon | E8 | -0.11 | -0.28 | 0.05 | 0.1748 |
| Shannon | NE1 | 0.02 | -0.08 | 0.12 | 0.7268 |
| Faith's Phylogenetic Diversity | E1 | -0.13 | -0.31 | 0.05 | 0.1695 |
| Faith's Phylogenetic Diversity | E2 | -0.06 | -0.27 | 0.14 | 0.5383 |
| Faith's Phylogenetic Diversity | E3 | -0.13 | -0.34 | 0.07 | 0.1923 |
| Faith's Phylogenetic Diversity | E4 | -0.07 | -0.19 | 0.05 | 0.2688 |
| Faith's Phylogenetic Diversity | E5 | 0.04 | -0.08 | 0.17 | 0.4977 |
| Faith's Phylogenetic Diversity | E6 | -0.03 | -0.15 | 0.09 | 0.6077 |
| Faith's Phylogenetic Diversity | E7 | -0.02 | -0.17 | 0.13 | 0.7920 |
| Faith's Phylogenetic Diversity | E8 | 0.10 | -0.06 | 0.27 | 0.2245 |
| Faith's Phylogenetic Diversity | NE1 | -0.03 | -0.14 | 0.07 | 0.5101 |
| Evenness | E1 | -0.19 | -0.37 | -0.01 | 0.0396 |
| Evenness | E2 | -0.07 | -0.27 | 0.13 | 0.5112 |
| Evenness | E3 | -0.12 | -0.32 | 0.08 | 0.2324 |
| Evenness | E4 | -0.01 | -0.13 | 0.11 | 0.8449 |
| Evenness | E5 | 0.05 | -0.07 | 0.17 | 0.4070 |
| Evenness | E6 | -0.04 | -0.15 | 0.08 | 0.5544 |
| Evenness | E7 | -0.12 | -0.27 | 0.03 | 0.1206 |
| Evenness | E8 | -0.15 | -0.31 | 0.02 | 0.0761 |
| Evenness | NE1 | 0.04 | -0.06 | 0.14 | 0.4693 |

* Model1:

Linear regression: Diversity index ~ β1 Group + β2 age + β3 sex + β4 BMI + β5 use of hypoglycemic or hypolipidemic medications + ϵ. The no-exposed control group (NE2, born in 1962-1964) was used as the reference group. The comparable groups including the the no-exposed group (NE1, born after 1964), three in utero exposed groups (E1-E3, born in 1959, 1960, and 1961, respectively), infancy- and toddler-exposed group (E4, born in 1956-1958), preschooler exposed group (E5, born in 1953-1955), school-aged child exposed group (E6, born in 1947-1952), adolescent exposed group (E7, born in 1942-1946) and adult exposed group (E8, born before 1942).

**Table S6. Association of famine exposure with gut microbial diversity in the CHNS cohort (Model 1)*.**

| **Outcome** | **Group** | **Adjusted beta** | **Lower confidence interval** | **Higher confidence interval** | **P value** |
| --- | --- | --- | --- | --- | --- |
| Observed OTUs | E1 | -0.32 | -0.58 | -0.06 | 0.0175 |
| Observed OTUs | E2 | 0.18 | -0.10 | 0.46 | 0.2139 |
| Observed OTUs | E3 | -0.29 | -0.60 | 0.01 | 0.0574 |
| Observed OTUs | E4 | -0.08 | -0.25 | 0.09 | 0.3589 |
| Observed OTUs | E5 | -0.12 | -0.29 | 0.05 | 0.1806 |
| Observed OTUs | E6 | -0.25 | -0.42 | -0.07 | 0.0064 |
| Observed OTUs | E7 | -0.50 | -0.73 | -0.26 | 0.0000 |
| Observed OTUs | E8 | -0.55 | -0.83 | -0.28 | 0.0001 |
| Observed OTUs | NE1 | -0.03 | -0.18 | 0.12 | 0.6877 |
| Shannon | E1 | -0.24 | -0.50 | 0.02 | 0.0734 |
| Shannon | E2 | 0.26 | -0.02 | 0.54 | 0.0650 |
| Shannon | E3 | -0.22 | -0.52 | 0.09 | 0.1594 |
| Shannon | E4 | 0.03 | -0.14 | 0.20 | 0.7008 |
| Shannon | E5 | 0.03 | -0.14 | 0.20 | 0.7657 |
| Shannon | E6 | -0.01 | -0.18 | 0.17 | 0.9363 |
| Shannon | E7 | -0.16 | -0.39 | 0.08 | 0.1989 |
| Shannon | E8 | -0.12 | -0.39 | 0.15 | 0.3840 |
| Shannon | NE1 | 0.02 | -0.12 | 0.17 | 0.7563 |
| Faith's Phylogenetic Diversity | E1 | -0.16 | -0.42 | 0.10 | 0.2226 |
| Faith's Phylogenetic Diversity | E2 | 0.25 | -0.03 | 0.53 | 0.0840 |
| Faith's Phylogenetic Diversity | E3 | -0.21 | -0.52 | 0.09 | 0.1683 |
| Faith's Phylogenetic Diversity | E4 | -0.03 | -0.20 | 0.14 | 0.7041 |
| Faith's Phylogenetic Diversity | E5 | -0.01 | -0.18 | 0.16 | 0.8691 |
| Faith's Phylogenetic Diversity | E6 | -0.14 | -0.32 | 0.03 | 0.1124 |
| Faith's Phylogenetic Diversity | E7 | -0.35 | -0.59 | -0.11 | 0.0041 |
| Faith's Phylogenetic Diversity | E8 | -0.39 | -0.66 | -0.12 | 0.0053 |
| Faith's Phylogenetic Diversity | NE1 | -0.02 | -0.17 | 0.13 | 0.8120 |
| Evenness | E1 | -0.11 | -0.37 | 0.15 | 0.4123 |
| Evenness | E2 | 0.27 | -0.02 | 0.55 | 0.0638 |
| Evenness | E3 | -0.14 | -0.45 | 0.16 | 0.3485 |
| Evenness | E4 | 0.10 | -0.07 | 0.27 | 0.2662 |
| Evenness | E5 | 0.10 | -0.07 | 0.27 | 0.2345 |
| Evenness | E6 | 0.13 | -0.05 | 0.30 | 0.1587 |
| Evenness | E7 | 0.08 | -0.16 | 0.32 | 0.5199 |
| Evenness | E8 | 0.15 | -0.12 | 0.42 | 0.2836 |
| Evenness | NE1 | 0.06 | -0.08 | 0.21 | 0.4032 |

* Model1:

Linear regression: Diversity index ~ β1Group + β2 age + β3 sex + β4 BMI + β5 use of hypoglycemic or hypolipidemic medications + ϵ. The no-exposed control group (NE2, born in 1962-1964) was used as the reference group. The comparable groups including the the no-exposed group (NE1, born after 1964), three in utero exposed groups (E1-E3, born in 1959, 1960, and 1961, respectively), infancy- and toddler-exposed group (E4, born in 1956-1958), preschooler exposed group (E5, born in 1953-1955), school-aged child exposed group (E6, born in 1947-1952), adolescent exposed group (E7, born in 1942-1946) and adult exposed group (E8, born before 1942).

**Table S7. Association of famine exposure with gut microbial diversity in the GNHS cohort (Model 2)*.**

| **Outcome** | **Group** | **Adjusted beta** | **Lower confidence interval** | **Higher confidence interval** | **P value** |
| --- | --- | --- | --- | --- | --- |
| Observed OTUs | E1 | -0.56 | -1.03 | -0.09 | 0.0196 |
| Observed OTUs | E2 | 0.03 | -0.57 | 0.62 | 0.9317 |
| Observed OTUs | E3 | -0.06 | -0.98 | 0.86 | 0.8949 |
| Observed OTUs | E4 | -0.26 | -0.64 | 0.12 | 0.1767 |
| Observed OTUs | E5 | -0.23 | -0.65 | 0.18 | 0.2674 |
| Observed OTUs | E6 | -0.27 | -0.76 | 0.21 | 0.2728 |
| Observed OTUs | E7 | -0.36 | -0.97 | 0.24 | 0.2426 |
| Observed OTUs | E8 | -0.52 | -1.26 | 0.21 | 0.1644 |
| Observed OTUs | NE1 | 0.30 | -0.15 | 0.75 | 0.1901 |
| Shannon | E1 | -0.56 | -1.04 | -0.09 | 0.0207 |
| Shannon | E2 | -0.11 | -0.71 | 0.49 | 0.7242 |
| Shannon | E3 | -0.04 | -0.96 | 0.89 | 0.9397 |
| Shannon | E4 | -0.23 | -0.61 | 0.15 | 0.2356 |
| Shannon | E5 | -0.21 | -0.63 | 0.20 | 0.3169 |
| Shannon | E6 | -0.20 | -0.69 | 0.29 | 0.4238 |
| Shannon | E7 | -0.30 | -0.91 | 0.32 | 0.3434 |
| Shannon | E8 | -0.30 | -1.05 | 0.44 | 0.4224 |
| Shannon | NE1 | 0.04 | -0.42 | 0.49 | 0.8747 |
| Faith's Phylogenetic Diversity | E1 | -0.42 | -0.89 | 0.06 | 0.0847 |
| Faith's Phylogenetic Diversity | E2 | 0.46 | -0.14 | 1.06 | 0.1365 |
| Faith's Phylogenetic Diversity | E3 | 0.28 | -0.65 | 1.21 | 0.5545 |
| Faith's Phylogenetic Diversity | E4 | -0.12 | -0.50 | 0.27 | 0.5510 |
| Faith's Phylogenetic Diversity | E5 | -0.09 | -0.51 | 0.32 | 0.6586 |
| Faith's Phylogenetic Diversity | E6 | -0.05 | -0.54 | 0.44 | 0.8347 |
| Faith's Phylogenetic Diversity | E7 | -0.12 | -0.74 | 0.49 | 0.6920 |
| Faith's Phylogenetic Diversity | E8 | -0.21 | -0.96 | 0.53 | 0.5776 |
| Faith's Phylogenetic Diversity | NE1 | 0.14 | -0.32 | 0.60 | 0.5464 |
| Eveness | E1 | -0.42 | -0.89 | 0.06 | 0.0866 |
| Eveness | E2 | -0.18 | -0.78 | 0.43 | 0.5639 |
| Eveness | E3 | -0.04 | -0.97 | 0.89 | 0.9296 |
| Eveness | E4 | -0.17 | -0.56 | 0.21 | 0.3719 |
| Eveness | E5 | -0.19 | -0.61 | 0.23 | 0.3825 |
| Eveness | E6 | -0.15 | -0.64 | 0.34 | 0.5560 |
| Eveness | E7 | -0.27 | -0.89 | 0.35 | 0.3893 |
| Eveness | E8 | -0.16 | -0.91 | 0.58 | 0.6678 |
| Eveness | NE1 | -0.13 | -0.59 | 0.33 | 0.5746 |

* Model2: Model1 + sequencing depth + dietary and lifestyle factors.

**Table S8. Association of famine exposure with gut microbial diversity in the GGMP cohort (Model 2)*.**

| **Outcome** | **Group** | **Adjusted beta** | **Lower confidence interval** | **Higher confidence interval** | **P value** |
| --- | --- | --- | --- | --- | --- |
| Observed OTUs | E1 | -0.18 | -0.37 | -0.0019 | 0.0476 |
| Observed OTUs | E2 | -0.07 | -0.27 | 0.13 | 0.4889 |
| Observed OTUs | E3 | -0.16 | -0.36 | 0.04 | 0.1195 |
| Observed OTUs | E4 | -0.10 | -0.22 | 0.02 | 0.1080 |
| Observed OTUs | E5 | 0.01 | -0.11 | 0.13 | 0.8901 |
| Observed OTUs | E6 | -0.06 | -0.18 | 0.06 | 0.3243 |
| Observed OTUs | E7 | -0.03 | -0.18 | 0.12 | 0.7193 |
| Observed OTUs | E8 | 0.04 | -0.13 | 0.20 | 0.6480 |
| Observed OTUs | NE1 | -0.05 | -0.16 | 0.05 | 0.3025 |
| Shannon | E1 | -0.20 | -0.38 | -0.02 | 0.0307 |
| Shannon | E2 | -0.06 | -0.26 | 0.14 | 0.5462 |
| Shannon | E3 | -0.14 | -0.34 | 0.06 | 0.1721 |
| Shannon | E4 | -0.04 | -0.16 | 0.08 | 0.5518 |
| Shannon | E5 | 0.05 | -0.07 | 0.17 | 0.4286 |
| Shannon | E6 | -0.04 | -0.16 | 0.08 | 0.5010 |
| Shannon | E7 | -0.10 | -0.25 | 0.05 | 0.2039 |
| Shannon | E8 | -0.10 | -0.26 | 0.07 | 0.2446 |
| Shannon | NE1 | 0.02 | -0.09 | 0.12 | 0.7666 |
| Faith's Phylogenetic Diversity | E1 | -0.11 | -0.29 | 0.07 | 0.2347 |
| Faith's Phylogenetic Diversity | E2 | -0.06 | -0.26 | 0.14 | 0.5558 |
| Faith's Phylogenetic Diversity | E3 | -0.13 | -0.33 | 0.08 | 0.2206 |
| Faith's Phylogenetic Diversity | E4 | -0.07 | -0.18 | 0.05 | 0.2845 |
| Faith's Phylogenetic Diversity | E5 | 0.05 | -0.07 | 0.17 | 0.4340 |
| Faith's Phylogenetic Diversity | E6 | -0.02 | -0.14 | 0.09 | 0.6992 |
| Faith's Phylogenetic Diversity | E7 | 0.00 | -0.15 | 0.15 | 0.9996 |
| Faith's Phylogenetic Diversity | E8 | 0.12 | -0.04 | 0.29 | 0.1413 |
| Faith's Phylogenetic Diversity | NE1 | -0.04 | -0.14 | 0.07 | 0.4738 |
| Eveness | E1 | -0.19 | -0.37 | -0.01 | 0.0436 |
| Eveness | E2 | -0.06 | -0.26 | 0.14 | 0.5577 |
| Eveness | E3 | -0.12 | -0.32 | 0.08 | 0.2515 |
| Eveness | E4 | -0.01 | -0.13 | 0.11 | 0.8658 |
| Eveness | E5 | 0.06 | -0.06 | 0.18 | 0.3547 |
| Eveness | E6 | -0.03 | -0.15 | 0.09 | 0.6171 |
| Eveness | E7 | -0.11 | -0.26 | 0.04 | 0.1490 |
| Eveness | E8 | -0.13 | -0.30 | 0.03 | 0.1150 |
| Eveness | NE1 | 0.04 | -0.07 | 0.14 | 0.4928 |

* Model2: Model1 + sequencing depth + dietary and lifestyle factors.

**Table S9. Association of famine exposure with gut microbial diversity in the CHNS cohort (Model 2)*.**

| **Outcome** | **Group** | **Adjusted beta** | **Lower confidence interval** | **Higher confidence interval** | **P value** |
| --- | --- | --- | --- | --- | --- |
| Observed OTUs | E1 | -0.27 | -0.52 | -0.02 | 0.0313 |
| Observed OTUs | E2 | 0.17 | -0.10 | 0.44 | 0.2078 |
| Observed OTUs | E3 | -0.15 | -0.44 | 0.14 | 0.3063 |
| Observed OTUs | E4 | -0.08 | -0.24 | 0.08 | 0.3484 |
| Observed OTUs | E5 | -0.06 | -0.22 | 0.11 | 0.4946 |
| Observed OTUs | E6 | -0.07 | -0.24 | 0.10 | 0.4083 |
| Observed OTUs | E7 | -0.16 | -0.39 | 0.07 | 0.1799 |
| Observed OTUs | E8 | -0.21 | -0.48 | 0.05 | 0.1137 |
| Observed OTUs | NE1 | -0.02 | -0.16 | 0.12 | 0.7622 |
| Shannon | E1 | -0.23 | -0.49 | 0.03 | 0.0818 |
| Shannon | E2 | 0.26 | -0.02 | 0.54 | 0.0685 |
| Shannon | E3 | -0.17 | -0.47 | 0.13 | 0.2724 |
| Shannon | E4 | 0.03 | -0.14 | 0.20 | 0.7349 |
| Shannon | E5 | 0.04 | -0.12 | 0.21 | 0.6059 |
| Shannon | E6 | 0.06 | -0.12 | 0.23 | 0.5446 |
| Shannon | E7 | -0.03 | -0.27 | 0.21 | 0.8171 |
| Shannon | E8 | 0.00 | -0.28 | 0.28 | 0.9915 |
| Shannon | NE1 | 0.02 | -0.12 | 0.17 | 0.7424 |
| Faith's Phylogenetic Diversity | E1 | -0.13 | -0.39 | 0.12 | 0.3088 |
| Faith's Phylogenetic Diversity | E2 | 0.24 | -0.03 | 0.51 | 0.0808 |
| Faith's Phylogenetic Diversity | E3 | -0.10 | -0.39 | 0.20 | 0.5238 |
| Faith's Phylogenetic Diversity | E4 | -0.03 | -0.20 | 0.13 | 0.7079 |
| Faith's Phylogenetic Diversity | E5 | 0.03 | -0.13 | 0.20 | 0.6913 |
| Faith's Phylogenetic Diversity | E6 | 0.00 | -0.18 | 0.17 | 0.9831 |
| Faith's Phylogenetic Diversity | E7 | -0.07 | -0.31 | 0.16 | 0.5553 |
| Faith's Phylogenetic Diversity | E8 | -0.11 | -0.38 | 0.16 | 0.4107 |
| Faith's Phylogenetic Diversity | NE1 | -0.01 | -0.16 | 0.13 | 0.8635 |
| Eveness | E1 | -0.12 | -0.38 | 0.14 | 0.3610 |
| Eveness | E2 | 0.26 | -0.02 | 0.54 | 0.0659 |
| Eveness | E3 | -0.15 | -0.46 | 0.15 | 0.3182 |
| Eveness | E4 | 0.09 | -0.08 | 0.26 | 0.3028 |
| Eveness | E5 | 0.10 | -0.07 | 0.27 | 0.2602 |
| Eveness | E6 | 0.12 | -0.06 | 0.30 | 0.1851 |
| Eveness | E7 | 0.07 | -0.17 | 0.32 | 0.5499 |
| Eveness | E8 | 0.14 | -0.14 | 0.42 | 0.3294 |
| Eveness | NE1 | 0.06 | -0.09 | 0.21 | 0.4291 |

* Model2: Model1 + sequencing depth + dietary and lifestyle factors.

**Table S10. Association of famine exposure with gut microbial diversity in the GNHS cohort (Model 3)*.**

| **Outcome** | **Group** | **Adjusted beta** | **Lower confidence interval** | **Higher confidence interval** | **P value** |
| --- | --- | --- | --- | --- | --- |
| Observed OTUs | E1 | -0.57 | -1.07 | -0.07 | 0.0251 |
| Observed OTUs | E2 | 0.06 | -0.56 | 0.67 | 0.8597 |
| Observed OTUs | E3 | -0.10 | -1.03 | 0.82 | 0.8232 |
| Observed OTUs | E4 | -0.33 | -0.73 | 0.07 | 0.1104 |
| Observed OTUs | E5 | -0.30 | -0.74 | 0.14 | 0.1871 |
| Observed OTUs | E6 | -0.33 | -0.85 | 0.19 | 0.2133 |
| Observed OTUs | E7 | -0.44 | -1.09 | 0.21 | 0.1872 |
| Observed OTUs | E8 | -0.71 | -1.50 | 0.09 | 0.0814 |
| Observed OTUs | NE1 | 0.35 | -0.13 | 0.83 | 0.1490 |
| Shannon | E1 | -0.54 | -1.05 | -0.04 | 0.0341 |
| Shannon | E2 | -0.09 | -0.72 | 0.53 | 0.7654 |
| Shannon | E3 | -0.07 | -0.99 | 0.86 | 0.8862 |
| Shannon | E4 | -0.27 | -0.67 | 0.14 | 0.1943 |
| Shannon | E5 | -0.26 | -0.70 | 0.18 | 0.2513 |
| Shannon | E6 | -0.22 | -0.74 | 0.30 | 0.3976 |
| Shannon | E7 | -0.34 | -1.00 | 0.31 | 0.3030 |
| Shannon | E8 | -0.40 | -1.20 | 0.39 | 0.3215 |
| Shannon | NE1 | 0.07 | -0.41 | 0.55 | 0.7771 |
| Faith's Phylogenetic Diversity | E1 | -0.41 | -0.92 | 0.10 | 0.1135 |
| Faith's Phylogenetic Diversity | E2 | 0.53 | -0.10 | 1.15 | 0.0986 |
| Faith's Phylogenetic Diversity | E3 | 0.22 | -0.71 | 1.16 | 0.6378 |
| Faith's Phylogenetic Diversity | E4 | -0.19 | -0.59 | 0.22 | 0.3658 |
| Faith's Phylogenetic Diversity | E5 | -0.14 | -0.59 | 0.30 | 0.5280 |
| Faith's Phylogenetic Diversity | E6 | -0.12 | -0.64 | 0.40 | 0.6534 |
| Faith's Phylogenetic Diversity | E7 | -0.20 | -0.86 | 0.45 | 0.5421 |
| Faith's Phylogenetic Diversity | E8 | -0.38 | -1.18 | 0.42 | 0.3548 |
| Faith's Phylogenetic Diversity | NE1 | 0.16 | -0.32 | 0.65 | 0.5038 |
| Eveness | E1 | -0.37 | -0.88 | 0.13 | 0.1490 |
| Eveness | E2 | -0.21 | -0.84 | 0.42 | 0.5154 |
| Eveness | E3 | -0.06 | -0.99 | 0.88 | 0.9065 |
| Eveness | E4 | -0.18 | -0.59 | 0.22 | 0.3811 |
| Eveness | E5 | -0.21 | -0.66 | 0.23 | 0.3472 |
| Eveness | E6 | -0.14 | -0.67 | 0.38 | 0.5935 |
| Eveness | E7 | -0.29 | -0.95 | 0.37 | 0.3965 |
| Eveness | E8 | -0.19 | -1.00 | 0.62 | 0.6458 |
| Eveness | NE1 | -0.12 | -0.60 | 0.37 | 0.6414 |

* Model3: Same as the model 1, but excluding the participants with type 2 diabetes.

**Table S11. Association of famine exposure with gut microbial diversity in the GGMP cohort (Model 3)*.**

| **Outcome** | **Group** | **Adjusted beta** | **Lower confidence interval** | **Higher confidence interval** | **P value** |
| --- | --- | --- | --- | --- | --- |
| Observed OTUs | E1 | -0.18 | -0.37 | 0.01 | 0.0644 |
| Observed OTUs | E2 | -0.12 | -0.33 | 0.09 | 0.2723 |
| Observed OTUs | E3 | -0.06 | -0.28 | 0.15 | 0.5541 |
| Observed OTUs | E4 | -0.04 | -0.17 | 0.08 | 0.4861 |
| Observed OTUs | E5 | 0.05 | -0.08 | 0.18 | 0.4432 |
| Observed OTUs | E6 | -0.06 | -0.18 | 0.06 | 0.3455 |
| Observed OTUs | E7 | 0.01 | -0.15 | 0.16 | 0.9187 |
| Observed OTUs | E8 | 0.05 | -0.12 | 0.22 | 0.5689 |
| Observed OTUs | NE1 | -0.04 | -0.15 | 0.06 | 0.4346 |
| Shannon | E1 | -0.17 | -0.36 | 0.02 | 0.0847 |
| Shannon | E2 | -0.13 | -0.35 | 0.08 | 0.2162 |
| Shannon | E3 | -0.12 | -0.34 | 0.09 | 0.2524 |
| Shannon | E4 | -0.01 | -0.13 | 0.11 | 0.8754 |
| Shannon | E5 | 0.08 | -0.05 | 0.21 | 0.2090 |
| Shannon | E6 | -0.04 | -0.16 | 0.08 | 0.5232 |
| Shannon | E7 | -0.01 | -0.17 | 0.14 | 0.8637 |
| Shannon | E8 | -0.10 | -0.27 | 0.07 | 0.2645 |
| Shannon | NE1 | 0.02 | -0.08 | 0.13 | 0.6767 |
| Faith's Phylogenetic Diversity | E1 | -0.14 | -0.33 | 0.05 | 0.1517 |
| Faith's Phylogenetic Diversity | E2 | -0.11 | -0.32 | 0.11 | 0.3363 |
| Faith's Phylogenetic Diversity | E3 | -0.07 | -0.28 | 0.14 | 0.5238 |
| Faith's Phylogenetic Diversity | E4 | -0.04 | -0.16 | 0.09 | 0.5854 |
| Faith's Phylogenetic Diversity | E5 | 0.07 | -0.05 | 0.20 | 0.2581 |
| Faith's Phylogenetic Diversity | E6 | -0.02 | -0.15 | 0.10 | 0.7006 |
| Faith's Phylogenetic Diversity | E7 | 0.04 | -0.12 | 0.20 | 0.6339 |
| Faith's Phylogenetic Diversity | E8 | 0.12 | -0.05 | 0.29 | 0.1660 |
| Faith's Phylogenetic Diversity | NE1 | -0.04 | -0.15 | 0.07 | 0.4485 |
| Eveness | E1 | -0.15 | -0.33 | 0.04 | 0.1219 |
| Eveness | E2 | -0.13 | -0.34 | 0.08 | 0.2264 |
| Eveness | E3 | -0.13 | -0.34 | 0.08 | 0.2215 |
| Eveness | E4 | 0.00 | -0.12 | 0.13 | 0.9583 |
| Eveness | E5 | 0.08 | -0.04 | 0.21 | 0.1886 |
| Eveness | E6 | -0.03 | -0.15 | 0.09 | 0.6269 |
| Eveness | E7 | -0.02 | -0.17 | 0.14 | 0.8354 |
| Eveness | E8 | -0.13 | -0.30 | 0.03 | 0.1178 |
| Eveness | NE1 | 0.04 | -0.06 | 0.15 | 0.4425 |

* Model3: Same as the model 1, but excluding the participants with type 2 diabetes.

**Table S12. Association of famine exposure with gut microbial diversity in the CHNS cohort (Model 3)*.**

| **Outcome** | **Group** | **Adjusted beta** | **Lower confidence interval** | **Higher confidence interval** | **P value** |
| --- | --- | --- | --- | --- | --- |
| Observed OTUs | E1 | -0.36 | -0.64 | -0.08 | 0.0115 |
| Observed OTUs | E2 | 0.21 | -0.08 | 0.50 | 0.1506 |
| Observed OTUs | E3 | -0.11 | -0.42 | 0.21 | 0.4997 |
| Observed OTUs | E4 | -0.06 | -0.23 | 0.12 | 0.5393 |
| Observed OTUs | E5 | -0.05 | -0.23 | 0.13 | 0.6122 |
| Observed OTUs | E6 | -0.08 | -0.27 | 0.10 | 0.3736 |
| Observed OTUs | E7 | -0.16 | -0.42 | 0.09 | 0.2017 |
| Observed OTUs | E8 | -0.19 | -0.48 | 0.09 | 0.1875 |
| Observed OTUs | NE1 | -0.03 | -0.18 | 0.12 | 0.6771 |
| Shannon | E1 | -0.30 | -0.58 | -0.01 | 0.0400 |
| Shannon | E2 | 0.38 | 0.09 | 0.68 | 0.0111 |
| Shannon | E3 | -0.12 | -0.45 | 0.20 | 0.4562 |
| Shannon | E4 | 0.05 | -0.13 | 0.23 | 0.6087 |
| Shannon | E5 | 0.06 | -0.12 | 0.24 | 0.5147 |
| Shannon | E6 | 0.11 | -0.08 | 0.29 | 0.2766 |
| Shannon | E7 | 0.01 | -0.25 | 0.27 | 0.9413 |
| Shannon | E8 | 0.08 | -0.22 | 0.37 | 0.6170 |
| Shannon | NE1 | 0.04 | -0.12 | 0.19 | 0.6181 |
| Faith's Phylogenetic Diversity | E1 | -0.19 | -0.47 | 0.09 | 0.1921 |
| Faith's Phylogenetic Diversity | E2 | 0.28 | -0.01 | 0.58 | 0.0580 |
| Faith's Phylogenetic Diversity | E3 | -0.07 | -0.39 | 0.25 | 0.6832 |
| Faith's Phylogenetic Diversity | E4 | 0.00 | -0.18 | 0.18 | 0.9906 |
| Faith's Phylogenetic Diversity | E5 | 0.05 | -0.13 | 0.23 | 0.5889 |
| Faith's Phylogenetic Diversity | E6 | -0.02 | -0.21 | 0.17 | 0.8534 |
| Faith's Phylogenetic Diversity | E7 | -0.09 | -0.35 | 0.17 | 0.5029 |
| Faith's Phylogenetic Diversity | E8 | -0.09 | -0.38 | 0.20 | 0.5467 |
| Faith's Phylogenetic Diversity | NE1 | -0.03 | -0.19 | 0.12 | 0.6547 |
| Eveness | E1 | -0.16 | -0.45 | 0.12 | 0.2620 |
| Eveness | E2 | 0.42 | 0.12 | 0.72 | 0.0055 |
| Eveness | E3 | -0.11 | -0.44 | 0.21 | 0.4874 |
| Eveness | E4 | 0.11 | -0.07 | 0.29 | 0.2484 |
| Eveness | E5 | 0.11 | -0.07 | 0.30 | 0.2288 |
| Eveness | E6 | 0.20 | 0.01 | 0.39 | 0.0384 |
| Eveness | E7 | 0.14 | -0.12 | 0.40 | 0.2768 |
| Eveness | E8 | 0.23 | -0.06 | 0.53 | 0.1243 |
| Eveness | NE1 | 0.08 | -0.07 | 0.24 | 0.2915 |

* Model3: Same as the model 1, but excluding the participants with type 2 diabetes.

**Table S13. Association of famine exposure with gut microbial diversity in the GGMP cohort (Model 4)*.**

| **Outcome** | **Group** | **Adjusted beta** | **Lower confidence interval** | **Higher confidence interval** | **P value** |
| --- | --- | --- | --- | --- | --- |
| Observed OTUs | E1 | -0.20 | -0.38 | -0.02 | 0.0325 |
| Observed OTUs | E2 | -0.10 | -0.30 | 0.11 | 0.3541 |
| Observed OTUs | E3 | -0.16 | -0.36 | 0.04 | 0.1172 |
| Observed OTUs | E4 | -0.10 | -0.22 | 0.02 | 0.0881 |
| Observed OTUs | E5 | 0.01 | -0.12 | 0.13 | 0.9206 |
| Observed OTUs | E6 | -0.08 | -0.20 | 0.05 | 0.2259 |
| Observed OTUs | E7 | -0.06 | -0.22 | 0.10 | 0.4751 |
| Observed OTUs | E8 | -0.01 | -0.19 | 0.16 | 0.8738 |
| Observed OTUs | NE1 | -0.03 | -0.10 | 0.04 | 0.3500 |
| Shannon | E1 | -0.20 | -0.38 | -0.02 | 0.0335 |
| Shannon | E2 | -0.07 | -0.27 | 0.13 | 0.5140 |
| Shannon | E3 | -0.12 | -0.32 | 0.08 | 0.2310 |
| Shannon | E4 | -0.02 | -0.14 | 0.10 | 0.6850 |
| Shannon | E5 | 0.07 | -0.06 | 0.19 | 0.3088 |
| Shannon | E6 | -0.03 | -0.15 | 0.10 | 0.6517 |
| Shannon | E7 | -0.08 | -0.24 | 0.08 | 0.3149 |
| Shannon | E8 | -0.09 | -0.27 | 0.09 | 0.3131 |
| Shannon | NE1 | 0.03 | -0.04 | 0.10 | 0.4353 |
| Faith's Phylogenetic Diversity | E1 | -0.13 | -0.31 | 0.05 | 0.1551 |
| Faith's Phylogenetic Diversity | E2 | -0.08 | -0.28 | 0.12 | 0.4317 |
| Faith's Phylogenetic Diversity | E3 | -0.14 | -0.34 | 0.06 | 0.1669 |
| Faith's Phylogenetic Diversity | E4 | -0.08 | -0.20 | 0.04 | 0.1889 |
| Faith's Phylogenetic Diversity | E5 | 0.04 | -0.08 | 0.17 | 0.5049 |
| Faith's Phylogenetic Diversity | E6 | -0.05 | -0.17 | 0.08 | 0.4619 |
| Faith's Phylogenetic Diversity | E7 | -0.04 | -0.20 | 0.12 | 0.5893 |
| Faith's Phylogenetic Diversity | E8 | 0.06 | -0.12 | 0.24 | 0.5143 |
| Faith's Phylogenetic Diversity | NE1 | -0.02 | -0.09 | 0.05 | 0.5683 |
| Eveness | E1 | -0.18 | -0.36 | 0.00 | 0.0527 |
| Eveness | E2 | -0.06 | -0.26 | 0.14 | 0.5636 |
| Eveness | E3 | -0.10 | -0.30 | 0.10 | 0.3413 |
| Eveness | E4 | 0.00 | -0.11 | 0.12 | 0.9384 |
| Eveness | E5 | 0.08 | -0.05 | 0.20 | 0.2348 |
| Eveness | E6 | -0.01 | -0.14 | 0.11 | 0.8547 |
| Eveness | E7 | -0.08 | -0.24 | 0.08 | 0.3044 |
| Eveness | E8 | -0.11 | -0.29 | 0.07 | 0.2218 |
| Eveness | NE1 | 0.04 | -0.03 | 0.11 | 0.2181 |

* Model4: Same as the model 1, but defined participants born after 1978 as the new reference group.

**Table S14. Association of famine exposure with gut microbial diversity in the CHNS cohort (Model 4)*.**

| **Outcome** | **Group** | **Adjusted beta** | **Lower confidence interval** | **Higher confidence interval** | **P value** |
| --- | --- | --- | --- | --- | --- |
| Observed OTUs | E1 | -0.28 | -0.54 | -0.02 | 0.0382 |
| Observed OTUs | E2 | 0.22 | -0.06 | 0.50 | 0.1315 |
| Observed OTUs | E3 | -0.25 | -0.56 | 0.05 | 0.0979 |
| Observed OTUs | E4 | -0.04 | -0.22 | 0.14 | 0.6440 |
| Observed OTUs | E5 | -0.08 | -0.26 | 0.10 | 0.3970 |
| Observed OTUs | E6 | -0.21 | -0.41 | -0.01 | 0.0361 |
| Observed OTUs | E7 | -0.46 | -0.72 | -0.20 | 0.0005 |
| Observed OTUs | E8 | -0.52 | -0.82 | -0.23 | 0.0005 |
| Observed OTUs | NE1 | 0.03 | -0.07 | 0.13 | 0.5524 |
| Shannon | E1 | -0.21 | -0.47 | 0.06 | 0.1247 |
| Shannon | E2 | 0.29 | 0.01 | 0.58 | 0.0397 |
| Shannon | E3 | -0.19 | -0.49 | 0.11 | 0.2219 |
| Shannon | E4 | 0.07 | -0.11 | 0.25 | 0.4481 |
| Shannon | E5 | 0.07 | -0.12 | 0.25 | 0.4825 |
| Shannon | E6 | 0.04 | -0.16 | 0.24 | 0.6990 |
| Shannon | E7 | -0.10 | -0.36 | 0.16 | 0.4438 |
| Shannon | E8 | -0.06 | -0.35 | 0.24 | 0.7008 |
| Shannon | NE1 | 0.05 | -0.04 | 0.15 | 0.2758 |
| Faith's Phylogenetic Diversity | E1 | -0.14 | -0.40 | 0.13 | 0.3022 |
| Faith's Phylogenetic Diversity | E2 | 0.27 | -0.01 | 0.55 | 0.0586 |
| Faith's Phylogenetic Diversity | E3 | -0.19 | -0.49 | 0.11 | 0.2207 |
| Faith's Phylogenetic Diversity | E4 | -0.01 | -0.19 | 0.17 | 0.9189 |
| Faith's Phylogenetic Diversity | E5 | 0.01 | -0.18 | 0.19 | 0.9239 |
| Faith's Phylogenetic Diversity | E6 | -0.12 | -0.32 | 0.08 | 0.2296 |
| Faith's Phylogenetic Diversity | E7 | -0.33 | -0.59 | -0.07 | 0.0134 |
| Faith's Phylogenetic Diversity | E8 | -0.37 | -0.66 | -0.07 | 0.0148 |
| Faith's Phylogenetic Diversity | NE1 | 0.02 | -0.08 | 0.12 | 0.6997 |
| Eveness | E1 | -0.08 | -0.35 | 0.18 | 0.5324 |
| Eveness | E2 | 0.29 | 0.01 | 0.57 | 0.0446 |
| Eveness | E3 | -0.13 | -0.43 | 0.18 | 0.4168 |
| Eveness | E4 | 0.13 | -0.05 | 0.31 | 0.1598 |
| Eveness | E5 | 0.14 | -0.04 | 0.33 | 0.1284 |
| Eveness | E6 | 0.18 | -0.02 | 0.37 | 0.0766 |
| Eveness | E7 | 0.14 | -0.11 | 0.40 | 0.2730 |
| Eveness | E8 | 0.23 | -0.06 | 0.53 | 0.1246 |
| Eveness | NE1 | 0.07 | -0.03 | 0.17 | 0.1653 |

* Model4: Same as the model 1, but defined participants born after 1978 as the new reference group.

**Table S15.** **Association of in utero famine exposed with gut microbial diversity in the three cohorts*.**

| **Outcome** | **Adjusted beta** | **Lower confidence interval** | **Higher confidence interval** | **P value** | **Cohort** |
| --- | --- | --- | --- | --- | --- |
| Observed OTUs | -0.18 | -0.49 | 0.12 | 0.2354 | GNHS |
| Shannon | -0.30 | -0.60 | 0.00 | 0.0530 | GNHS |
| Faith's Phylogenetic Diversity | -0.04 | -0.34 | 0.27 | 0.7985 | GNHS |
| Eveness | -0.29 | -0.60 | 0.01 | 0.0551 | GNHS |
| Observed OTUs | -0.10 | -0.21 | 0.02 | 0.1111 | GGMP |
| Shannon | -0.12 | -0.24 | 0.00 | 0.0419 | GGMP |
| Faith's Phylogenetic Diversity | -0.07 | -0.19 | 0.04 | 0.2220 | GGMP |
| Eveness | -0.12 | -0.25 | 0.00 | 0.0441 | GGMP |
| Observed OTUs | -0.11 | -0.27 | 0.06 | 0.2067 | CHNS |
| Shannon | -0.09 | -0.26 | 0.08 | 0.3110 | CHNS |
| Faith's Phylogenetic Diversity | -0.02 | -0.19 | 0.14 | 0.7666 | CHNS |
| Eveness | -0.05 | -0.22 | 0.12 | 0.5698 | CHNS |

*Linear regression: Diversity index ~ β1Group + β2 age + β3 sex + β4 BMI + β5 use of hypoglycemic or hypolipidemic medications + ϵ. The no-exposed control group (NE2, born in 1962-1964) was used as the reference group, and three in utero exposed groups (E1-E3, born in 1959, 1960, and 1961, respectively) were combined as the comparable group.

**Table S16. Association of famine exposure with gut microbial diversity after age balance*.**

| **Outcome** | **Group** | **Adjusted beta** | **Lower confidence interval** | **Higher confidence interval** | **P value** | **Cohort** |
| --- | --- | --- | --- | --- | --- | --- |
| Observed OTUs | E1 | -0.43 | -0.79 | -0.07 | 0.0206 | GNHS |
| Observed OTUs | E2 | 0.33 | -0.20 | 0.86 | 0.2222 | GNHS |
| Observed OTUs | E3 | -0.01 | -0.91 | 0.90 | 0.9912 | GNHS |
| Shannon | E1 | -0.49 | -0.85 | -0.13 | 0.0074 | GNHS |
| Shannon | E2 | 0.06 | -0.47 | 0.59 | 0.8220 | GNHS |
| Shannon | E3 | 0.02 | -0.88 | 0.92 | 0.9726 | GNHS |
| Faith's Phylogenetic Diversity | E1 | -0.35 | -0.71 | 0.01 | 0.0582 | GNHS |
| Faith's Phylogenetic Diversity | E2 | 0.57 | 0.05 | 1.10 | 0.0333 | GNHS |
| Faith's Phylogenetic Diversity | E3 | 0.32 | -0.58 | 1.22 | 0.4858 | GNHS |
| Eveness | E1 | -0.39 | -0.75 | -0.03 | 0.0351 | GNHS |
| Eveness | E2 | -0.17 | -0.69 | 0.36 | 0.5365 | GNHS |
| Eveness | E3 | 0.00 | -0.90 | 0.90 | 0.9957 | GNHS |
| Observed OTUs | E1 | -0.14 | -0.31 | 0.04 | 0.1212 | GGMP |
| Observed OTUs | E2 | -0.02 | -0.21 | 0.18 | 0.8554 | GGMP |
| Observed OTUs | E3 | -0.12 | -0.31 | 0.08 | 0.2338 | GGMP |
| Shannon | E1 | -0.19 | -0.37 | -0.01 | 0.0377 | GGMP |
| Shannon | E2 | -0.04 | -0.24 | 0.16 | 0.6789 | GGMP |
| Shannon | E3 | -0.12 | -0.32 | 0.07 | 0.2183 | GGMP |
| Faith's Phylogenetic Diversity | E1 | -0.09 | -0.27 | 0.09 | 0.3216 | GGMP |
| Faith's Phylogenetic Diversity | E2 | -0.02 | -0.22 | 0.17 | 0.8155 | GGMP |
| Faith's Phylogenetic Diversity | E3 | -0.10 | -0.30 | 0.09 | 0.2969 | GGMP |
| Eveness | E1 | -0.19 | -0.37 | -0.01 | 0.0401 | GGMP |
| Eveness | E2 | -0.05 | -0.25 | 0.15 | 0.5978 | GGMP |
| Eveness | E3 | -0.11 | -0.31 | 0.09 | 0.2689 | GGMP |
| Observed OTUs | E1 | -0.28 | -0.52 | -0.04 | 0.0246 | CHNS |
| Observed OTUs | E2 | 0.23 | -0.03 | 0.50 | 0.0818 | CHNS |
| Observed OTUs | E3 | -0.27 | -0.55 | 0.02 | 0.0669 | CHNS |
| Shannon | E1 | -0.28 | -0.53 | -0.03 | 0.0283 | CHNS |
| Shannon | E2 | 0.26 | -0.01 | 0.53 | 0.0615 | CHNS |
| Shannon | E3 | -0.23 | -0.52 | 0.07 | 0.1282 | CHNS |
| Faith's Phylogenetic Diversity | E1 | -0.15 | -0.39 | 0.09 | 0.2108 | CHNS |
| Faith's Phylogenetic Diversity | E2 | 0.28 | 0.02 | 0.53 | 0.0353 | CHNS |
| Faith's Phylogenetic Diversity | E3 | -0.20 | -0.48 | 0.08 | 0.1554 | CHNS |
| Eveness | E1 | -0.19 | -0.45 | 0.07 | 0.1445 | CHNS |
| Eveness | E2 | 0.22 | -0.06 | 0.50 | 0.1172 | CHNS |
| Eveness | E3 | -0.18 | -0.48 | 0.13 | 0.2534 | CHNS |

*Linear regression: Diversity index ~ β1Group + β2 age + β3 sex + β4 BMI + β5 use of hypoglycemic or hypolipidemic medications + ϵ. The new reference group represents the combination of participants born in 1962-1964 (NE2, unexposed control group) and 1956-1958 (E4, infancy and toddler exposed group), and three in utero exposed groups (E1-E3) were used as as the comparable groups.

**Table S17.** **Shifts in the gut microbial composition relative to the no-exposed control group in the three cohorts*.**

| **PCOA** | **Group** | **Adjusted beta** | **Lower confidence interval** | **Higher confidence interval** | **P value** | **Cohort** |
| --- | --- | --- | --- | --- | --- | --- |
| PCOA1 | NE1 | 0.12 | -0.34 | 0.58 | 0.6069 | GNHS |
| PCOA1 | E1 | 0.49 | 0.01 | 0.97 | 0.0440 | GNHS |
| PCOA1 | E2 | 0.15 | -0.45 | 0.76 | 0.6176 | GNHS |
| PCOA1 | E3 | 0.06 | -0.88 | 0.99 | 0.9055 | GNHS |
| PCOA1 | E4 | 0.16 | -0.22 | 0.54 | 0.4090 | GNHS |
| PCOA1 | E5 | 0.08 | -0.33 | 0.50 | 0.6978 | GNHS |
| PCOA1 | E6 | 0.08 | -0.41 | 0.57 | 0.7562 | GNHS |
| PCOA1 | E7 | 0.00 | -0.61 | 0.62 | 0.9923 | GNHS |
| PCOA1 | E8 | 0.20 | -0.54 | 0.95 | 0.5961 | GNHS |
| PCOA2 | NE1 | -0.16 | -0.62 | 0.30 | 0.4915 | GNHS |
| PCOA2 | E1 | -0.22 | -0.69 | 0.26 | 0.3662 | GNHS |
| PCOA2 | E2 | -0.29 | -0.89 | 0.31 | 0.3412 | GNHS |
| PCOA2 | E3 | 0.24 | -0.69 | 1.16 | 0.6165 | GNHS |
| PCOA2 | E4 | -0.10 | -0.48 | 0.27 | 0.5897 | GNHS |
| PCOA2 | E5 | -0.13 | -0.54 | 0.29 | 0.5493 | GNHS |
| PCOA2 | E6 | -0.08 | -0.56 | 0.41 | 0.7556 | GNHS |
| PCOA2 | E7 | -0.23 | -0.84 | 0.38 | 0.4622 | GNHS |
| PCOA2 | E8 | -0.14 | -0.88 | 0.61 | 0.7191 | GNHS |
| PCOA1 | NE1 | 0.05 | -0.05 | 0.15 | 0.3377 | GGMP |
| PCOA1 | E1 | -0.04 | -0.22 | 0.14 | 0.6742 | GGMP |
| PCOA1 | E2 | -0.14 | -0.34 | 0.06 | 0.1694 | GGMP |
| PCOA1 | E3 | -0.01 | -0.21 | 0.19 | 0.9147 | GGMP |
| PCOA1 | E4 | -0.02 | -0.14 | 0.10 | 0.7142 | GGMP |
| PCOA1 | E5 | 0.02 | -0.10 | 0.14 | 0.7514 | GGMP |
| PCOA1 | E6 | 0.04 | -0.08 | 0.15 | 0.5282 | GGMP |
| PCOA1 | E7 | 0.07 | -0.08 | 0.21 | 0.3808 | GGMP |
| PCOA1 | E8 | 0.12 | -0.05 | 0.28 | 0.1634 | GGMP |
| PCOA2 | NE1 | 0.00 | -0.10 | 0.11 | 0.9518 | GGMP |
| PCOA2 | E1 | -0.18 | -0.36 | 0.00 | 0.0560 | GGMP |
| PCOA2 | E2 | -0.10 | -0.30 | 0.10 | 0.3384 | GGMP |
| PCOA2 | E3 | -0.05 | -0.25 | 0.15 | 0.6286 | GGMP |
| PCOA2 | E4 | -0.04 | -0.16 | 0.08 | 0.5222 | GGMP |
| PCOA2 | E5 | -0.02 | -0.15 | 0.10 | 0.6909 | GGMP |
| PCOA2 | E6 | -0.04 | -0.16 | 0.07 | 0.4668 | GGMP |
| PCOA2 | E7 | -0.12 | -0.27 | 0.03 | 0.1141 | GGMP |
| PCOA2 | E8 | -0.26 | -0.42 | -0.09 | 0.0022 | GGMP |
| PCOA1 | NE1 | -0.15 | -0.29 | 0.00 | 0.0492 | CHNS |
| PCOA1 | E1 | 0.26 | 0.00 | 0.52 | 0.0496 | CHNS |
| PCOA1 | E2 | -0.02 | -0.30 | 0.26 | 0.8894 | CHNS |
| PCOA1 | E3 | 0.03 | -0.28 | 0.33 | 0.8681 | CHNS |
| PCOA1 | E4 | 0.05 | -0.12 | 0.22 | 0.5344 | CHNS |
| PCOA1 | E5 | 0.01 | -0.16 | 0.18 | 0.8842 | CHNS |
| PCOA1 | E6 | 0.06 | -0.12 | 0.23 | 0.5319 | CHNS |
| PCOA1 | E7 | 0.40 | 0.16 | 0.64 | 0.0010 | CHNS |
| PCOA1 | E8 | 0.39 | 0.12 | 0.66 | 0.0052 | CHNS |
| PCOA2 | NE1 | 0.07 | -0.08 | 0.21 | 0.3817 | CHNS |
| PCOA2 | E1 | -0.47 | -0.73 | -0.21 | 0.0003 | CHNS |
| PCOA2 | E2 | -0.07 | -0.35 | 0.21 | 0.6327 | CHNS |
| PCOA2 | E3 | -0.21 | -0.51 | 0.09 | 0.1725 | CHNS |
| PCOA2 | E4 | 0.02 | -0.15 | 0.19 | 0.7908 | CHNS |
| PCOA2 | E5 | -0.11 | -0.28 | 0.06 | 0.1860 | CHNS |
| PCOA2 | E6 | -0.23 | -0.41 | -0.05 | 0.0103 | CHNS |
| PCOA2 | E7 | -0.20 | -0.43 | 0.04 | 0.1056 | CHNS |
| PCOA2 | E8 | -0.39 | -0.67 | -0.12 | 0.0044 | CHNS |

* Linear regression: PCOA index ~ β1Group + β2 age + β3 sex + β4 BMI + β5 use of hypoglycemic or hypolipidemic medications + ϵ. The no-exposed control group (NE2, born in 1962-1964) was used as the reference group. The comparable groups including the the no-exposed group (NE1, born after 1964), three in utero exposed groups (E1-E3, born in 1959, 1960, and 1961, respectively), infancy and toddler exposed group (E4, born in 1956-1958), preschooler exposed group (E5, born in 1953-1955), school-aged child exposed group (E6, born in 1947-1952), adolescent exposed group (E7, born in 1942-1946) and adult exposed group (E8, born before 1942).
